# Mechanistic Study of the Conductance and Enhanced Single-Molecule Detection in a Polymer–Electrolyte Nanopore

**DOI:** 10.1021/acsnanoscienceau.2c00050

**Published:** 2023-01-10

**Authors:** Fabio Marcuccio, Dimitrios Soulias, Chalmers C. C. Chau, Sheena E. Radford, Eric Hewitt, Paolo Actis, Martin Andrew Edwards

**Affiliations:** † School of Electronic and Electrical Engineering, University of Leeds, LeedsLS2 9JT, U.K.; ‡ Bragg Centre for Materials Research, University of Leeds, LeedsLS2 9JT, U.K.; § School of Molecular and Cellular Biology and Astbury Centre for Structural Molecular Biology, University of Leeds, LeedsLS2 9JT, U.K.; ∥ Department of Chemistry and Biochemistry, University of Arkansas, Fayetteville, Arkansas72701, United States

**Keywords:** nanopipette, nanopore, finite-element modeling, nanofluidic diode, DNA, poly(ethylene) glycol, PEG

## Abstract

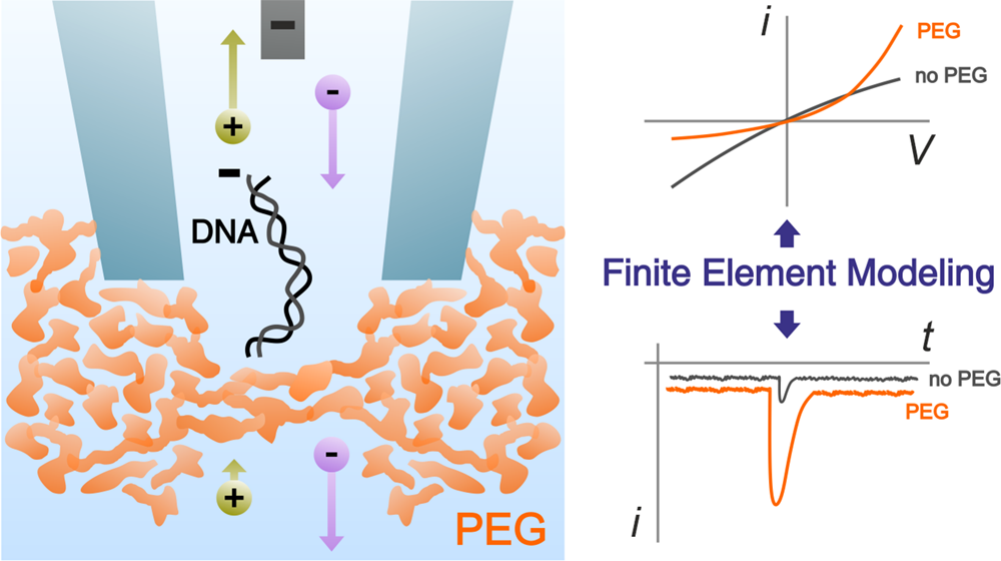

Solid-state nanopores have been widely employed in the detection of biomolecules, but low signal-to-noise ratios still represent a major obstacle in the discrimination of nucleic acid and protein sequences substantially smaller than the nanopore diameter. The addition of 50% poly(ethylene) glycol (PEG) to the external solution is a simple way to enhance the detection of such biomolecules. Here, we demonstrate with finite-element modeling and experiments that the addition of PEG to the external solution introduces a strong imbalance in the transport properties of cations and anions, drastically affecting the current response of the nanopore. We further show that the strong asymmetric current response is due to a polarity-dependent ion distribution and transport at the nanopipette tip region, leading to either ion depletion or enrichment for few tens of nanometers across its aperture. We provide evidence that a combination of the decreased/increased diffusion coefficients of cations/anions in the bath outside the nanopore and the interaction between a translocating molecule and the nanopore–bath interface is responsible for the increase in the translocation signals. We expect this new mechanism to contribute to further developments in nanopore sensing by suggesting that tuning the diffusion coefficients of ions could enhance the sensitivity of the system.

## Introduction

Nanopore sensing is one of the leading label-free techniques for the analysis and manipulation of single molecules due to its high throughput and sensitivity.^1−4^ In nanopore measurements, an ionic current is generated by applying a potential between two electrodes placed in two reservoirs separated by a small orifice. In general, the translocation of an analyte through a nanopore causes a decrease in magnitude of the ionic current due to the restricted transport of ions through the orifice (resistive-pulse event).^1^ The amplitude, duration, and shape of the translocation event provide important information about the physicochemical properties of the molecule, such as size, charge, and shape.^1,5,6^ Under low electrolyte concentrations, charged molecules, such as double-stranded DNA (dsDNA), can lead to a local ion enrichment, resulting in a current enhancement (conductive-pulse event).^1,7^ The origin of this current enhancement was initially attributed to the additional charge carried by the counterion cloud around the dsDNA molecule.^8^ However, conductive events are also observed at high salt concentrations,^9,10^ suggesting that the total ionic current can increase or decrease depending on the concentration and transport properties of ions in and around the nanopore.^7,11,12^

Despite the developments in the field over the past decades,^13^ the application of solid-state nanopores for the detection of proteins and short nucleic acids still remains challenging, requiring nanopores of comparable size to the molecules (<10 nm diameter), which are difficult to fabricate reproducibly.^14^ Furthermore, the nanopore system needs to have a high signal-to-noise ratio (SNR)^15^ to detect small perturbations to the ion current caused by the translocation of molecules and high-bandwidth electronics to characterize rapid translocations with sufficient temporal resolution.^16,17^ So far, finite-element modeling has been extensively used to examine electrokinetic phenomena in nanopores.^10,18−21^ In such systems, the ion current is due to the transport of ionic species under the influence of an electric field, and its physics can be considerably more complex than the one regulating simple ohmic conductors.^22^ For example, the charge on the nanopore wall induces an electric double layer, leading to non-uniform ion concentration distributions, and the interacting physics of ion transport, electric fields, and fluid flows result in a wide range of non-linear behavior.^19,23,24^

We have recently reported the enhanced single-molecule detection by a nanopore when 50% poly(ethylene) glycol (PEG) is added to the external solution,^25^ and we further characterized the system showing that the polymer–electrolyte interactions in the external solution govern the translocation dynamics of the analyte that is correlated to the properties of the electrolyte only in the external solution.^26^ Here, we present a mechanism explaining this enhancement by using a combination of experiments and multiphysics modeling. We developed a finite-element model by coupling Nernst–Planck and Poisson equations to describe the physics of ion transport under an applied electric field when a nanopore sensing experiment is carried out in the presence of 50% PEG. Based on the cation-binding properties of PEG that have been previously reported in the literature, our model assumes a decrease in the diffusion coefficients of cations relative to anions in the external solution.^27−31^ The model reproduces the experimental current–voltage responses in the presence and absence of PEG and provides an insight into the ion concentrations and transport rates responsible for the observed behavior. We then prove that the increase in the translocation signal is a combination of the unequal diffusion coefficients of ions in the bath outside the nanopore and the interaction between a translocating molecule and the nanopore–PEG interface. We expect this new mechanism to inform further developments in nanopore sensing by suggesting that approaches that affect the diffusion coefficients of ions in the external bath could be used to enhance the sensitivity of the system.

## Results and Discussion

**Figure 1:**
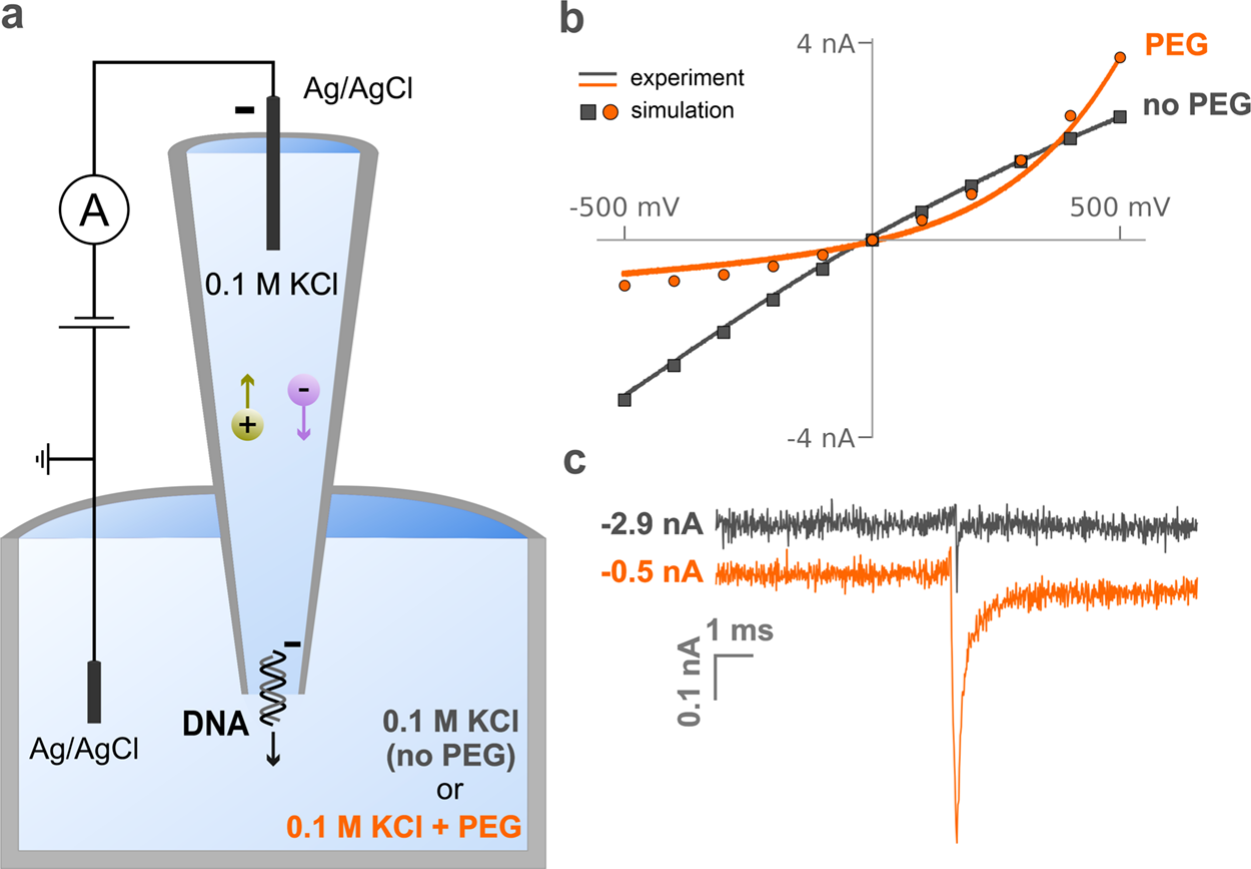
Schematic and representative data for current-voltage and conductive-pulse measurements of dsDNA translocation through a nanopipette. (a) Nanopipette (25 nm pore diameter), filled with a 0.3 nM solution of 4.8 kbp dsDNA in 0.1 M KCl, is immersed in a solution of the same electrolyte with and without the presence of 50% (w/v) PEG 35K. The application of a negative potential to a Ag/AgCl quasi-reference electrode inside the nanopipette with respect to a ground electrode in the external solution causes outbound migration of DNA molecules. (b) Experimental (curves) and simulated (points) voltammograms of the nanopipette in the presence (orange) and absence (gray) of PEG in the outside solution. (c) Representative current trace recorded upon translocation of a dsDNA molecule through the nanopipette aperture with (orange trace) and without (gray trace) the presence of PEG in the external solution. Further examples of voltammetry and current traces from this and other nanopipettes are included in the Supporting Information (Section S4).

Figure 1a shows the experimental setup used throughout this work in which a model solid-state nanopore based on a quartz nanopipette (aperture 25 nm in diameter) filled with a 0.1 M KCl solution is immersed into a bath containing 0.1 M KCl with or without 50% (w/v) PEG. In nanopore measurements, the current–voltage (*i–V*) response characterizes the ion transport, indirectly providing information about the physical properties of the nanopore (size, shape, and surface charge). The gray line in Figure 1b shows the current–voltage response of a nanopipette filled with a 0.1 M KCl solution and immersed in a bath containing 0.1 M KCl (no PEG). The slightly higher conductivity observed at a negative bias applied versus a positive bias is termed ion-current rectification (ICR) and arises from the negatively charged glass wall of the nanopipette, which makes the aperture region permselective to cations; this effect has been extensively described in the literature.^24,32−34^

When the same nanopipette is immersed in a bath of 0.1 M KCl with 50% PEG, a dramatic reversal in the rectification is observed in the *i–V* curve (orange line). The PEG solution is ∼9 times less conductive than 0.1 M KCl (Table ST1.2, Supporting Information), and, counterintuitively, the ion current observed at +500 mV is greater than the one measured in a PEG-free bath (above-bulk conductivity). Also, under a negative bias, the ion current is ∼4 times lower than observed without PEG in the external solution. This response cannot be explained only considering the difference in conductivity between the two solutions, or as a rectification effect induced by the surface charge on the nanopore wall, indicating that a different mechanism is responsible for the observed *i–V* response. We also demonstrated that the viscosity of the solution is not responsible for this observed phenomenon, as the *i–V* response obtained with PEG cannot be reproduced with other viscous solutions such as 50% (v/v) glycerol (Section S3, Supporting Information). In the following section, we describe a numerical model to calculate ionic currents (points in Figure 1b) in the PEG system and to explain this anomalous current–voltage behavior. The reproducibility of the experimental voltammograms in the presence and absence of PEG in the external solution was determined by testing six nanopipettes (Figures SF4.1 and SF4.2, Supporting Information).

As we have previously reported,^25^ the presence of PEG in the external solution leads to a 4-fold enhancement of the ion current observed when a single molecule translocates through the nanopore (Figures 1c and SF4.3, Supporting Information). In particular, the SNR in the presence of PEG in the external solution was found to be approximately 2.2 times larger than the SNR in the absence of PEG with values SNR_mean_^PEG^ = 26.4 ± 3.9 and SNR_mean_^no PEG^ = 11.9 ± 3.9 (Figure SF4.4, Section S4.4, Supporting Information). Interestingly, the signal in the presence of PEG does not quickly return to the original baseline level after the translocation peak, suggesting a different kinetics regulating the translocation dynamics. The same signals plotted over an extended period are shown in Figure SF4.8 (Section S4.7, Supporting Information). It is worth noting that as the two current traces displayed in Figure 1c were both recorded using the same nanopipette tip aperture (*d*= 25 nm), applied voltage (−500 mV), and composition of the inner solution (0.1 M KCl and 0.3 nM 4.8 kbp dsDNA), the observed enhancement is only driven by the presence of PEG in the external solution. Moreover, these traces are representative of a large (>1000) number of peaks recorded from a single measurement, and small fluctuations in the recorded values (i.e., current peak maximum and frequency of events) are expected due to potential variability in the electrolyte or nanopipette properties (i.e., temperature, concentration, pore geometry, and aperture size).^35^ For the PEG condition, individual translocation events randomly selected from recordings using three nanopipettes are shown in Figure SF4.3 (Supporting Information).

On another note, this experiment was repeated without adding any analyte to the nanopipette solution to check whether the conductive events were generated by the PEG molecules translocating through the pore. No translocation events were detected (Section S4.5, Figure SF4.5, Supporting Information), confirming that the conductive events were generated by the dsDNA molecules. To check whether the presence of PEG could change the properties of translocation events over time, a continuous 20 min measurement of dsDNA translocation was carried out. The analysis (Section S4.6, Supporting Information) shows that there are no significant changes over time in both the number of translocation events (Figure SF4.6, Supporting Information) and the translocation peak characteristics (Figure SF4.7, Supporting Information). During a conventional dsDNA translocation measurement through a nanopore where the solution is identical in both reservoirs, the conductive events are generally attributed to the presence of the counterion cloud carried by the dsDNA molecule and altered ion transport at the nanopipette tip, which result in a temporary increase in the ion concentration in this region.^7,10,11,36,37^ The following sections present a new mechanism in the nanopore systems that explains not only the anomalous *i–V* response related to PEG but also the enhanced single-molecule sensitivity.

### Finite-Element Simulations

We developed a finite-element model that coupled ion transport (diffusion and electromigration) and electrostatics at different applied potentials. A detailed description of the model is given in the Supporting Information. Briefly, a two-dimensional axisymmetric model simulates the geometry of a nanopipette as a simplified truncated hollow cone immersed in a spherical bath (Figure SF1.1, Supporting Information). The model was informed by experimental measurements (scanning electron microscopy graphs of the nanopipette tip geometry in Figure SF2.1, bulk conductivities and viscosities of the solutions in Table ST1.2, Supporting Information). The inner half-cone angle (θ), surface charge of the quartz glass (σ), and diffusion coefficients of the ions in the bath containing 50% PEG 35K (*D*_K_^*+*^, *D*_Cl_^*–*^) could not be directly measured experimentally. The inner half-cone angle (θ = 7°) was determined by comparing experiments with an analytical expression for the resistance of the nanopipette immersed in a 0.1 M KCl solution (see Section S2.2, Supporting Information for more details). Similarly, the surface charge at the nanopipette quartz wall  was estimated using the closest fit to the experimental current rectification data (Section S2.4, Supporting Information).

In our system, charge is carried by ions migrating due to the presence of an electric field (electromigration) and concentration gradient (diffusion).^23^ In 0.1 M KCl, the ion flux generated by electromigration depends on the sum of the diffusion coefficients of ions in solution (Section S2.3, Supporting Information), which defines the solution conductivity (κ) according to the Nernst−Einstein equation:
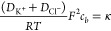
1where *D*_K^+^_ and *D*_Cl^–^_ are the diffusion coefficients of potassium and chloride, respectively, *c_b_* is the bulk concentration, *F* is the Faraday constant, *R* is the natural gas constant, and *T* is the temperature. For KCl, in normal conditions (no PEG), the ratio between the diffusion coefficients of the two species is very close to unity , meaning that the contribution of potassium and chloride to the total conductivity κ is approximately the same.

Evidence in the literature has shown that PEG associates with cations in solution.^28−31,38^ Zhang et al.^27^ developed a molecular dynamics model and proved the interaction between cations and PEG, finding that the trapping time of the ion in the polymer chain is highly dependent on the ion radius with longer trapping times for larger radii. These findings clearly indicate that the diffusion properties of cations in solution are affected by the presence of PEG. In the simulations, we considered this effect by assuming a change in the diffusion coefficients of the two ion species in the external solution. The properties of the 0.1 M KCl electrolyte inside the nanopipette were kept constant as described above.

We performed a parametric study to determine the ratio of the diffusion coefficients by decreasing the contribution of the potassium ion and increasing that of chloride  to the total conductivity κ_PEG_ (Section S2.3, Supporting Information) to describe the experimental *i–V* of the nanopipette in the presence of PEG, as shown in Figure 1b (orange curve). The study revealed that the lower the ratio of diffusion coefficients, the more asymmetric the *i–V* response will be (Figure SF2.3, Supporting Information), which supports our hypothesis that the polymer–cation interactions are responsible for the distinctive current response in the presence of PEG.^25,26^ We obtained the closest fit to the experimental data (orange square points, Figure 1b) by selecting a diffusion coefficient ratio of  = 0.54, meaning a 35% contribution from the cations and 65% from the anions to the total conductivity of the PEG solution. The simulated currents shown in Figure 1b (orange data points) quantitatively reproduce the experimentally observed *i*–*V* response (orange curve).

It is worth clarifying that all input parameters, with or without PEG in the external solution, were either measured experimentally (electrical conductivity, fluid viscosity, and electrolyte concentration) (Table ST1.2, Supporting Information) or found in the literature. In addition, we found that the nanopipette surface charge and any fluid flow in the system minimally influence the simulated *i*–*V* response in the presence of PEG in the external solution (Section 2.5 and Table ST2.1, Supporting Information); thus, all modeling results related to PEG presented below were obtained without considering these factors.

### Average Ion Concentration at the Tip Region

**Figure 2:**
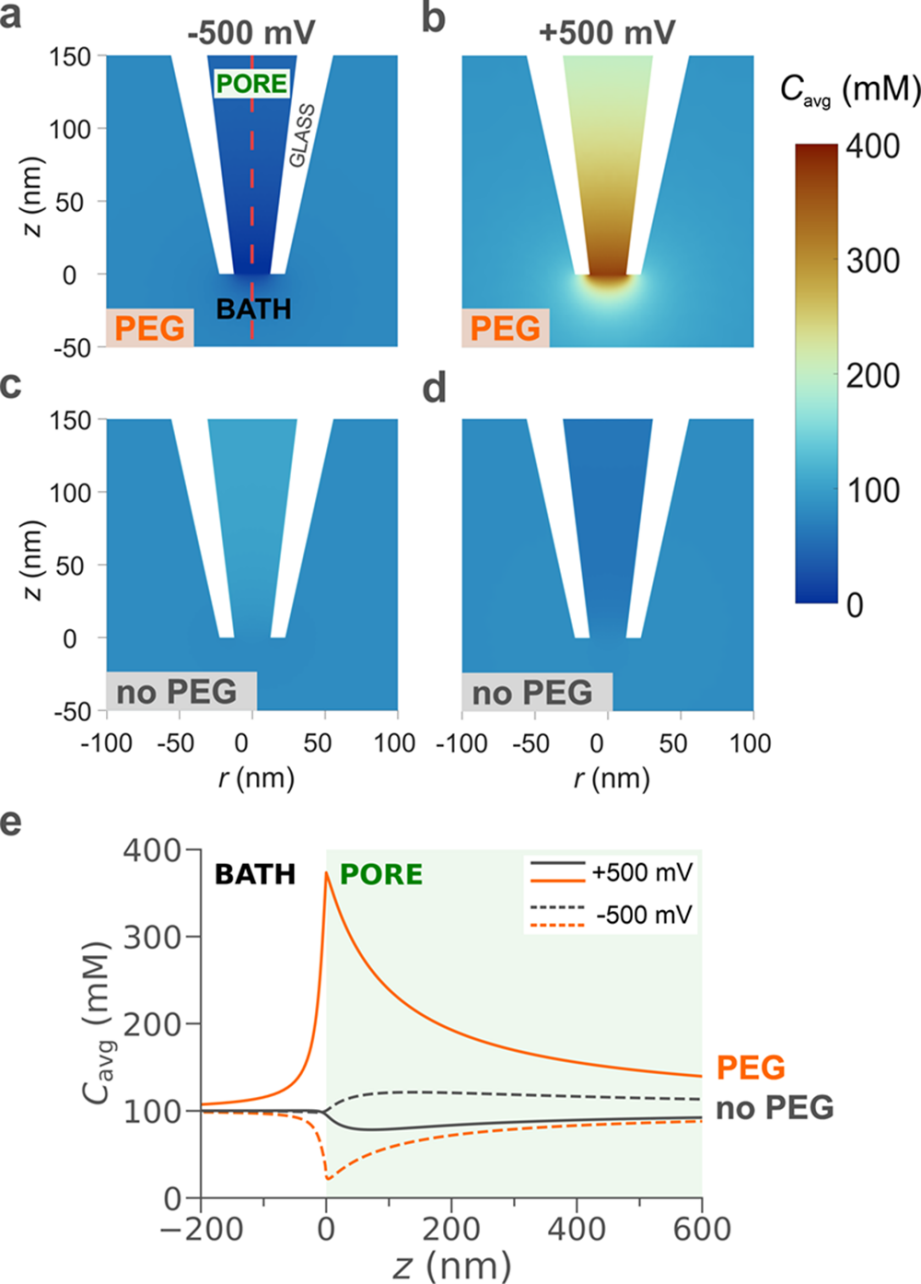
Simulated ion distributions close to the nanopipette tip at ±500 mV in the presence and absence of PEG in the external solution. Average concentration (*C*_avg_ = 1/2([K^+^] + [Cl^–^])) with (a, b) and without (c, d) PEG in the external solution for an applied voltage of (a, c) −500 mV and (b, d) 500 mV. (e) Average ion concentrations along the nanopipette axis of symmetry (red dashed line in panel a) in the presence (orange) and absence (gray) of PEG for negative (dashed curves) and positive (solid curves) bias applied. Note that average ion concentrations under different applied potentials and individual cation and anion concentration distributions are included in the Supporting Information (Section S5).

Figure 2 shows the average ion concentration  obtained with finite-element modeling under two opposite voltages applied (*V* = ±500 mV) in the presence (Figure 2a,b) and absence (Figure 2c,d) of PEG in the external solution (Section S5, Supporting Information). In the presence of PEG, a pronounced ion depletion is observed for *V* = –500 mV (Figure 2a), while ion enrichment is noticeable when *V* = +500 mV (Figure 2b), with a 20-fold increase in the ion concentration compared to when a negative bias is applied. This observation is the origin of the asymmetric current response observed in the presence of PEG (Figure 1b). In the absence of PEG in the external solution, a slightly higher ion concentration can be observed within the pore region under *V* = –500 mV (Figure 2c) compared to the case with *V* = +500 mV (Figure 2d). This explains the slightly asymmetric curve (ICR) for the no PEG case (gray curve) shown in Figure 1b.^24^Figure 2e plots the average ion concentration along the symmetry axis of the nanopipette (dashed red line, Figure 2a), allowing for quantitative comparison of the simulations. The average concentration for the PEG (orange curve) and no PEG (gray curve) cases is plotted for *V* = –500 mV (dashed line) and *V* = +500 mV (solid line). The average concentration under all the simulated applied potentials is shown in Figure SF5.2 (Section S5.2, Supporting Information). In our reference system, the interface between the nanopipette and the external solution is positioned at *z* = 0 nm, while *z* > 0 nm corresponds to the axis of symmetry inside the nanopipette and *z* < 0 nm to the external solution (Figure SF1.1, Supporting Information). Interestingly, the maximum ion concentration for *V* = +500 mV in the presence of PEG (orange solid line) is approximately four times higher than the corresponding case with no PEG (gray solid line). This observation indicates that the above-bulk conductivity arises from a dramatic increase in the ion concentration at the tip region of the nanopipette, despite the external solution in the presence of PEG being nine times less conductive (Table ST1.2, Supporting Information). It is worth noting that both the ion enrichment and depletion peaks at *z* = 0 nm approach *C*_avg_ = 100 mM when lower voltages are applied (±400, ±300, ±200, and ±100 mV), as illustrated in Figure SF5.2 (Section S5.2, Supporting Information).

Experimentally, a similar increase in conductivity is observed upon the translocation of a single dsDNA molecule in the presence of PEG in the external solution, as shown in Figure 1c, suggesting that the signal amplification is related to the number of ions in the sensing region of the nanopipette. The vast difference in the ion concentration between the positive and negative bias is similar to the behavior of nanofluidic diodes^39−43^ for ultrashort conical nanopores. In these studies, nanofluidic diodes were developed by introducing a surface charge discontinuity on a nanochannel, which forms a junction similar to bipolar semiconductors. In our case, we achieve a similar behavior by introducing an interface between a region where the values for the diffusion coefficients for cations and anions are approximately the same (i.e., the inner solution) and a region where the diffusion coefficient for the cations is much smaller than the one for anions due to the presence of PEG (i.e., the external solution). This discontinuity not only affects the ion distribution but also ion transport, as we describe in the next section.

### Ion Transport at the Tip Region

**Figure 3:**
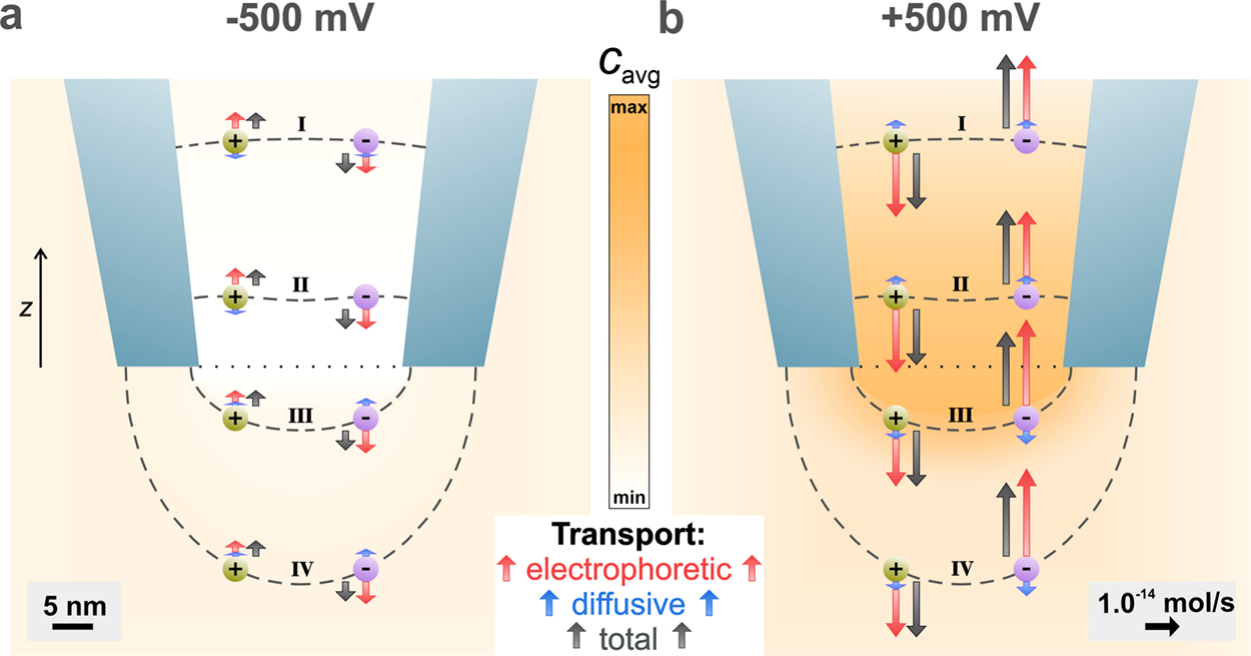
Visualization of the relative contributions of different physical processes to the transport rates of K^+^ and Cl^–^ at −500 mV (a) and +500 mV (b) with PEG in the outer solution. The lengths of the arrows represent the magnitude of the total transport rate (black) across the respective equipotential line (dashed gray: I, II, III, and IV), which is the sum of electrophoretic (red) and diffusive (blue) contributions. In addition, the arrows being parallel to the *z* axis and the ion positions were selected for illustration purposes only. Arrows for negligible diffusive contributions are not shown in the plot for ease of representation. The color map in the background represents the average ion concentration, and the dotted line at the nanopipette aperture represents the interface between the inner and the external solution. Further details on the transport calculations and the individual values for the transport rate of each ion for each boundary are included in the Supporting Information (Section S6).

The origin of the significant differences in the ion concentration (*C*_avg_) in the presence of PEG can be understood by a careful analysis of the ion transport (*N*_K^+^_, *N*_Cl^–^_) across the interface close to the nanopipette tip aperture, which represents the most sensitive region of our system^44^ (Section S6, Supporting Information). We define the “sensing region” as a region between two equipotential lines (dashed lines I and IV, Figure 3) where a 50% drop of the applied voltage is observed. In the case of −500 mV being applied, the voltage drop across the sensing region (Δ*V*_sens_) is equal to 250 mV. In the presence of PEG and under −500 mV, we found that this region is about 40 nm in length along the *z* axis (from *z* = –20 to *z* = 20 nm with the interface between the inner and external solutions set at *z* = 0) (Figures SF6.3 and SF6.4, Supporting Information).

This clearly indicates a highly resistive region positioned at the nanopipette tip, which leads to a significant drop in the measured current magnitude (baseline current), as shown in Figure 1b (orange curves and square points).

In any enclosed volume, the flux of ions through the surface surrounding the volume is equal to the rate of change in the number of ions (mass and charge conservation).^45^ The transport rate for each ion species (*N_i_*) was calculated by integrating the total flux of K^+^ and Cl^–^ separately, along the equipotential lines (dashed gray lines I, II, III, and IV, Figure 3) selected around the nanopipette tip. An extensive description of these calculations is provided in Section S6 of the Supporting Information. In a nutshell, for 0.1 M KCl, where both ion species have a valence of *z_i_* = 1, the difference between the number of charges (ions) entering and exiting each dashed line over time is proportional to the current.

Since no convection was considered for this simulation, the total ion transport rate (black arrow, Figure 3) can be broken down into two components, the electrophoretic (*N_i_^m^*) and diffusive (*N_i_^d^*) (red and blue arrows, respectively, Figure 3). Figure 3 illustrates all these three components, for both cations (green sphere) and anions (purple sphere), at four equipotential lines to highlight the marked difference in ion transport between the inner and outer solutions for *V* = ±500 mV. The total ion transport rate (black arrows) of each ion species for each applied potential remains constant across the designed dashed lines, verifying that mass and charge are conserved in the system and that the sum of the electrophoretic and diffusive components will always be the same. Based on the polarity of the applied voltage, cations/anions will get attracted/repelled resulting in electrophoretic ion transport either in or out of the nanopipette tip (dotted black line, Figure 3). Additionally, any gradients in the ion concentration (color map in the background of Figure 3) give rise to diffusive ion transport with both species moving toward (with *V* = −500 mV) or away from the tip interface (with *V* = 500 mV).

Figure 3 shows that the total ion transport rate at −500 mV is lower than the rate at 500 mV by 75%, which is in agreement with the experimental and simulated *i*–*V* responses presented in Figure 1b. It is important to note that the electrophoretic transport dominates diffusion in all cases. To summarize, when *V* = 500 mV, there are a larger number of ions flowing across the nanopipette tip aperture over time, which results in a higher current magnitude (Table ST6.2, Supporting Information), demonstrating that an asymmetric ion mobility is responsible for the observed above-bulk conductivity. In contrast, when *V* = −500 mV, there is a low number of ions flowing across the nanopipette tip aperture over time, resulting in a much lower current magnitude (Table ST6.1, Supporting Information), which again is consistent with the experimental data. Figure 3 shows that PEG acts as an anion-selective membrane in the external solution. When *V* = −500 mV, the cations in the nanopipette flow away from the sensing region toward the inner electrode, while cations in the external solution flow with a lower transport rate toward the nanopipette aperture. This creates a depletion region. On the contrary, when *V* = + 500 mV, the cations in the nanopipette flow from the inner electrode to the nanopipette tip, while cations at the nanopipette tip flow to the bath solution with hindered ion transport, creating an ion-enriched region.

### Mechanism of Current Enhancement upon dsDNA Translocation

DNA molecules carry a negative surface charge and form counterion clouds when immersed in electrolyte solutions (0.1 M KCl).^7,46^ In standard conditions (no PEG) and under negative potentials (−500 mV), the temporary increase in the current magnitude recorded during dsDNA translocation is due to a combination of the additional ions carried by the molecule to the sensing region of the nanopipette and the temporary change in the concentration and transport properties of ions in solution, which results in a temporary higher conductivity.^7^

In the presence of PEG, the physics related to the generated current upon dsDNA translocation through the nanopipette aperture is considerably more complex. Our previous work on the polymer–electrolyte nanopore showed that the single-molecule capture physics is remarkably different. In PEG, the capture of DNA follows a linear relationship between the molecule count and the applied voltage, suggesting a diffusion-limited regime in contrast to a barrier-limited regime in the absence of PEG.^26^ As previously explained, the nanopipette shows a remarkable ion depletion at the tip region with very few ions transporting through the interface when −500 mV is applied (see the ion concentrations in Figure 2a and transport in Figure 3a), while the external solution is mainly populated by anions, with cation transport hindered by intercalation in the PEG molecules.^27^ In these conditions, the counterion cloud carried by the dsDNA molecule certainly contributes to the temporal increase of the ion concentration, and thus the conductivity, of the system. However, this is not sufficient to explain the drastic current enhancement recorded experimentally. In fact, the charge carried by the translocating dsDNA molecule is the same regardless of the presence or absence of PEG in the external solution; thus, the increased conductivity should be approximately equal in both cases (see Section S7.1, Supporting Information).

**Figure 4:**
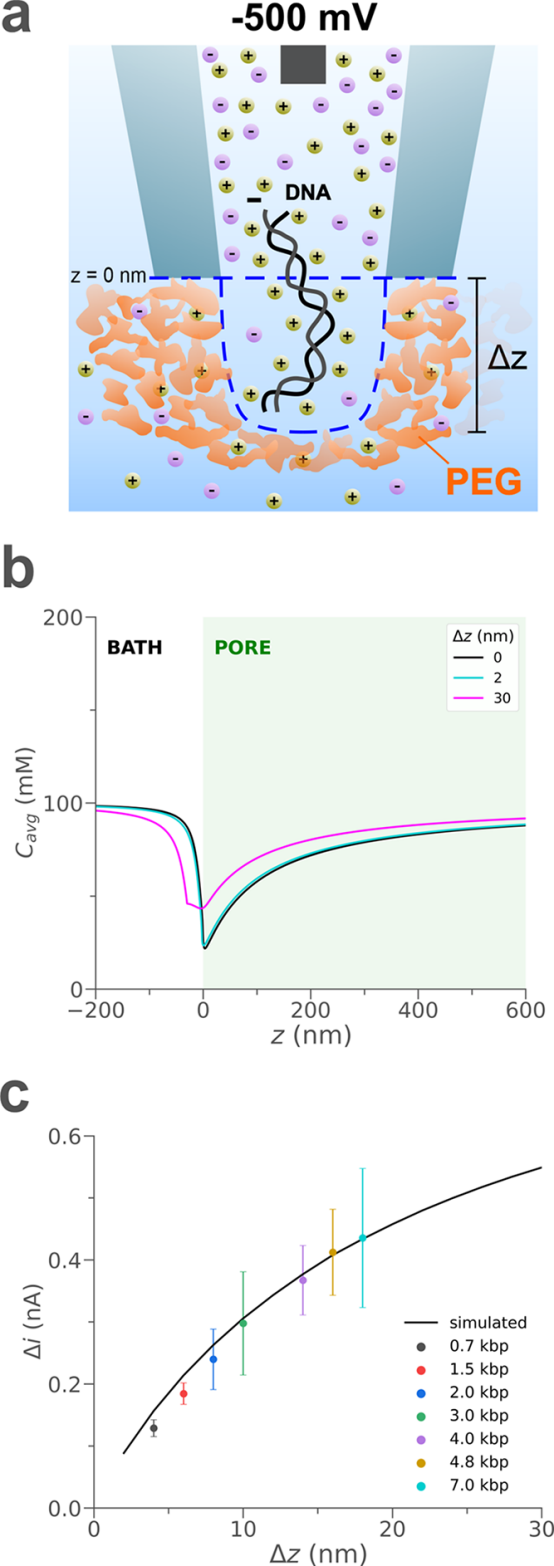
Proposed mechanism of current enhancement upon translocation of a dsDNA molecule. (a) Translocation of a dsDNA molecule through the nanopipette causes a temporary displacement of the interface (*Δz*) between the pore and external solution (blue dashed line), which results in a temporary ion enrichment in the nanopipette tip region (note that the illustrations are not in scale, and geometries were chosen for illustration purposes only). (b) Simulated average ion concentration along the axis of symmetry (*r* = 0 nm) for 0 nm (black), 2 nm (cyan), and 30 nm (magenta) interface displacements. (c) Simulated (black curve) and experimental (colored points) current peak maxima (Δ*i*) for different interface displacements toward the external solution and sizes of dsDNA molecules translocating through the nanopipette tip aperture toward the bath, respectively. The error bars represent the standard deviation of the experimental current peak maxima values. The horizontal coordinate of experimental data points was chosen according to the expected Δ*z* (Table ST7.1, Supporting Information). Further details on the interface displacement simulations and the experimental translocation data are included in the Supporting Information (Section S7).

We explored whether the mechanical interactions between dsDNA and PEG molecules at the interface of the internal and external solutions could temporarily alter the ion concentrations at the tip region. Briefly, we considered a rectangular protrusion of the domain inside the nanopipette (inner solution) toward the bath domain (external solution) to get a simplistic model of the interface shift caused by the translocation of dsDNA, as shown in Figure 4a. We performed a parametric study by varying the size of this protrusion (Δ*z*) from *z* = 0 to *z* = –30 nm with 2 nm steps. Figure 4b presents the simulated average ion concentration along the symmetry axis for three different interface displacements (0, 2, and 30 nm). As the interface moves further away from the nanopipette tip opening (*z* = 0 nm), the number of ions in the nanopipette’s sensing region increases, resulting in an enhanced current value. We found that an interface displacement of 16 nm toward the external solution is sufficient to cause an increase in the ion current to match the current peak maxima measured experimentally for the translocation of a single 4.8 kbp dsDNA molecule (Sections S7.2 and S7.3, Supporting Information). This current enhancement is due to a 33% increase in the ion concentration in the nanopipette sensing region (0 < *z* < 20 nm) caused by this shift in the interface.

To summarize, we found that the translocation of dsDNA molecules through the pore causes a temporary displacement of the interface, which results in a shift of the ion depleted region toward the bath. The consequence is ion enrichment in the sensing region inside the nanopipette, which results in higher conductivity and thus higher measured currents (Figure 4a). We speculate that the time required for the ion concentrations in the nanopipette sensing region to return to the pre-translocation values is at the origin of the longer time required to restore the initial baseline current (Figures 1c and SF4.8, Supporting Information). Note that in our simulations, we simplistically assume that the interface between the pore and the external solution without DNA is a straight line at *z* = 0 nm (no mixing, blue dashed line in Figure 4). The interface is likely to be more diffuse than represented in the model. However, the balancing of the fluxes shown in Figure 3 must still ultimately occur beyond the transition region. Thus, while the model does not capture the detail at the interface exactly, it does capture the reason. Using a more sophisticated model for the interface would certainly improve the accuracy of our calculations but not the level of our understanding of the system.

Based on this mechanism, we expect various dsDNA molecule sizes to have different effects on the translocation current, as recently reported by Confederat et al.^47^ for DNA origami. For instance, longer dsDNA molecules would displace the interface further toward the external solution. To test this hypothesis, we repeated the same experiment as the one illustrated in Figure 1 using a range of sizes of dsDNA as the analyte (0.7–7 kbp) with and without PEG in the outside bath (Figure 4c and Sections S7.3 and S7.4, Supporting Information). In PEG, experimental current peak maxima for the translocation of dsDNA molecules with sizes from 0.7 up to 7 kbp are in close agreement with the trend obtained from the simulated current values due to interface displacements, as shown in Figure 4c and Table ST7.1 in the Supporting Information. In the no PEG case, not only there is no evident correlation, but the detection is limited to molecules with a minimum size of 4.8 kbp (Figure SF7.2c, Supporting Information). These findings confirm our initial hypothesis that the current enhancement in the presence of PEG 35K upon dsDNA translocation cannot be explained only in terms of additional ions carried by the analyte, supporting the new pore-centric theory recently reported by Lastra et al.^22^ for a system based on a pore’s flux imbalance, but a mechanical interaction between the analyte and PEG molecules at the nanopipette tip opening must be taken into account.

To further support this, we experimentally verified that the voltammetric responses and current enhancement caused by PEG disappear when a positive pressure is applied at the back of the nanopipette to force PEG molecules away from the tip opening (Section S7.5, Supporting Information). This result shows that the PEG effect is completely canceled by disrupting the interface, underpinning the importance of the latter to the observed current enhancement.

## Conclusions

To summarize, we developed a finite-element model to improve our understanding of the dramatic current enhancement observed upon dsDNA molecule translocation through a nanopipette to an external solution containing 50% (w/v) PEG 35K. This system was successfully simulated by assuming a decrease/increase in the diffusion coefficients of cations/anions, respectively, due to the cation-binding properties of PEG. We observed that the characteristic *i*–*V* response in the presence of PEG is due to voltage-dependent ion concentrations at the tip region with ion enrichment at positive and ion depletion at negative potentials. A similar behavior was noticed in the asymmetric transport rates for each ion species across the tip orifice, resulting in higher currents at a positive applied bias compared to negative. Furthermore, we demonstrated that conventional mechanisms of current enhancement based on additional ions carried by the analyte are not sufficient to fully explain our system. Hence, we proposed a novel mechanism supported by experimental evidence, which relies on mechanical interactions between the translocating analyte and the interface between the solutions. We proved that such interactions could lead to alteration of the ion distribution at the tip orifice, which can result in temporary current increases. We expect that this work can provide a new paradigm in nanopore sensing, where the alteration of the ion transport properties of the external solution can be harnessed to provide enhanced SNRs allowing for the biochemical and structural analysis of proteins and other biomolecules.

## Materials and Methods

### Nanopipette Fabrication

Quartz capillaries of 1.0 mm outer diameter and 0.5 mm inner diameter (QF100-50-7.5; Sutter Instrument) were used to fabricate the nanopipette using the SU-P2000 laser puller (World Precision Instruments). A two-line protocol was used, line 1: HEAT 750/FIL 4/VEL 30/DEL 150/PUL 80, followed by line 2: HEAT 625/FIL 3/VEL 40/DEL 135/PUL 150. The pulling protocol is instrument-specific, and there is variation between different SU-P2000 pullers.

### External Bath Preparation

To generate 10 mL of the 50% (w/v) PEG 35K (Sigma Aldrich; 94646) 0.1 M KCl solution, 1 mL of a 1 M KCl solution, 4 mL of ddH_2_O, and 5 g of PEG 35K were mixed inside a tube. The tube was then left inside a 70 °C incubator for 2 h, followed by overnight incubation at 37 °C. The tubes were then left on a bench for 4 h to reach room temperature prior to use. All electrolytes were stored at room temperature.

### Double-Stranded DNA Preparation

To prepare the individual dsDNA samples, the GeneRuler 1 kbp plus DNA Ladder (SM1331: Thermo Fisher) was separated via a 0.8% agarose gel. The individual bands (0.7, 1.5, 2, 3, 4, 4.8, and 7 kbp) were physically isolated with a blade, and the dsDNA was extracted using the Monarch DNA Gel Extraction Kit according to the manufacturer specifications (T1020; New England Biolabs Inc.). The extracted dsDNA was further purified using the Genomic Clean and Concentrator Kit (D4010; Zymo Research). All dsDNA was eluted in the Monarch DNA Elution Buffer (T1016L; New England BioLabs Inc.) and stored at −20 °C. All the dsDNA was then diluted from stock to 0.3 nM with 0.1 M KCl.

### Ion Current Trace Recording

The nanopipettes were all filled with 0.3 nM dsDNA diluted in 0.1 M KCl (P/4240/60; Fisher Scientific) and fitted with a Ag/AgCl working electrode. The nanopipettes were immersed into the electrolyte bath containing or not containing PEG 35K with a Ag/AgCl reference electrode. The ionic current trace was recorded using a MultiClamp 700B patch-clamp amplifier (Molecular Devices) in voltage-clamp mode. The signal was filtered using a low-pass filter at 20 kHz and digitized with Digidata 1550B at a 100 kHz sampling rate and recorded using the software pClamp 10 (Molecular Devices).

### Finite-Element Modeling

Finite-element simulations were performed with COMSOL Multiphysics 5.6 (COMSOL Inc.).

## Data Availability

The IV curves, ion current traces, and simulation COMSOL report, input parameters and definition, geometry, physics, boundary conditions, and mesh modeling files associated with this paper are openly available from the University of Leeds data repository at https://doi.org/10.5518/1274.

## Supporting Information Available

Supporting Information contents: simulation overview and verification; definition of simulation input parameters; influence of external solution viscosity; reproducibility of experimental data; ion concentrations at the nanopipette tip region; ion transport and the definition of the sensing region; interface displacement model; and effect of an externally applied pressure on the voltammogram and dsDNA translocation (PDF)

## Abbreviation

dsDNA: double-stranded DNA
PEG: poly(ethylene) glycol
SF: supporting figure
SE: supporting equation
ST: supporting table
