# Supplementary material for: Mechanistic Study of the Conductance and Enhanced Single-Molecule Detection in a Polymer–Electrolyte Nanopore
Source: ACS Nanosci Au. 2023 Jan 10;3(2):172–81. doi: 10.1021/acsnanoscienceau.2c00050 (PMC10119975; doi:10.1021/acsnanoscienceau.2c00050)
Supplement: Supplementary file 1 — ng2c00050_si_001.pdf [file ng2c00050_si_001.pdf]

Supporting Information:

Mechanistic Study of the Conductance and Enhanced Single-Molecule Detection in a Polymer Electrolyte Nanopore

Fabio Marcuccio[1,2,‡], Dimitrios Soulias[1,2,‡], Chalmers C. C. Chau[1,2,3], Sheena E. Radford[3], Eric Hewitt[3], Paolo Actis[1,2,], Martin Andrew Edwards[4, *]*

[1] School of Electronic and Electrical Engineering, University of Leeds, Leeds, LS2 9JT, UK

[2] Bragg Centre for Materials Research, University of Leeds, Leeds, LS2 9JT, UK

[3] School of Molecular and Cellular Biology and Astbury Centre for Structural Molecular Biology, University of Leeds, Leeds, LS2 9JT, UK

[4] Department of Chemistry and Biochemistry, University of Arkansas, Fayetteville, AR, 72701, USA

‡ *These authors contributed equally to this work.*

* *Corresponding authors:* p.actis@leeds.ac.uk , maedw@uark.edu

Contents

S1 Simulation overview and verification 4

S1.1 Physics used for finite-element modelling 4

S1.2 Geometry and boundaries 5

S1.3 Boundary conditions and key numerical parameters 6

S1.4 Mesh 7

S1.5 Ion current measurement 8

S2 Definition of simulation input parameters 9

S2.1 Nanopipette pore diameter 9

S2.2 Analytical determination of inner half cone angle 10

S2.3 Determination of ion diffusion coefficients 12

S2.4 Determination of the surface charge on the nanopipette walls 14

S2.5 Evidence of negligible influence of surface charge and fluid flow 15

S3 Influence of external solution viscosity in the voltammogram 16

S3.1 Experimental voltammogram with 50% glycerol in the external solution 16

S3.2 Experimental conductivity and viscosity measurements 17

S4 Reproducibility of experimental data 18

S4.1 Voltammograms with three different nanopipettes in the presence of PEG 18

S4.2 Voltammograms with three different nanopipettes in absence of PEG 18

S4.3 Translocation current recordings with three different nanopipettes 19

S4.4 Signal-to-noise (SNR) ratio with and without PEG in the external solution 20

S4.5 Current recordings with no analyte in the nanopipette with PEG in the external solution 21

S4.6 Continuous measurement of dsDNA translocation into the PEG solution 22

S4.7 Translocation traces over an extended period. 23

S5 Ion concentrations at the nanopipette tip region 24

S5.1 Individual cation and anion concentrations along the symmetry axis 24

S5.2 Average ion concentrations along the symmetry axis under different applied potentials for the PEG condition 25

S6 Ion transport at the tip region and definition of sensing region 26

S6.1 Boundaries for the calculation of ion transport 26

S6.2 Calculating the transport rates of each ion species at the boundaries 27

S6.3 Defining the "sensing region" based on the electric potential distribution 28

S6.4 Transport rates of each ion species in the sensing region 30

S7 Mechanism of current enhancement upon dsDNA translocation 32

S7.1 Estimating the number of ions carried by single dsDNA in the "sensing region" 32

S7.2 Model for interface displacement due to dsDNA translocation 33
S7.3 Effect of dsDNA size on translocation current 34
S7.4 Agarose gel electrophoresis of dsDNA samples 36
S7.5 Effect of an externally applied pressure to the nanopipette 37
References 39

S1 Simulation overview and verification

S1.1 Physics used for finite-element modelling

In this work, we designed a finite-element model, based on a simplified geometry of a nanopipette tip (truncated cone) immersed in an electrolyte solution containing 50% (w/v) PEG, to solve the coupled Nernst-Planck and Poisson equations and understand the physical mechanisms involved. By allocating the appropriate boundary conditions on this two-dimensional (2D) axisymmetric model (Figure SF1.1), we determined the ion concentration and voltage distributions in the fluid. For the solution of these equations, we assumed steady-state conditions.

The Nernst-Planck equation ("*Transport of Diluted Species*, *chds*") describes the ion transport properties based on the diffusive and electrophoretic fluxes, as follows[1]:

$$\vec{J_i} = -D_i^{\alpha}\nabla c_i + \frac{z_i F}{RT} D_i^{\alpha} c_i \nabla V \qquad \text{(SE1.1)},$$

where $\vec{J_i}$: ion flux, D_i^{α}: diffusion coefficient of ion i in phase α (PEG/no PEG), c_i: ion concentration, z_i: ion valence number, F: Faraday constant, R: universal gas constant, T: temperature, and V: voltage.

The Poisson equation ("*Electrostatics*, *es*") describes how the electric potential is related to the ion concentration:

$$\nabla^2 V = -\frac{F}{\varepsilon}\sum_i z_i c_i \qquad \text{(SE1.2)},$$

where ε: medium electric permittivity.

S1.2 Geometry and boundaries

Figure SF1.1 illustrates the two-dimensional axisymmetric geometry of the finite-element model, including three key boundaries for solving equations SE1.1 and SE1.2. The nanopipette tip aperture radius (r) is 12.5 nm, quartz wall thickness (t_w) is 10 nm, inner half-cone angle (θ) is 7° and the pore length (L) is 50 µm. The top boundary (1) represents the bottom surface of the Ag/AgCl electrode immersed in the nanopipette where voltage is applied. Boundary 2 is quartz glass walls allocated with a surface charge (σ), and the semi-circular boundary at the bottom (3) represents the surface of the ground electrode (Ag/AgCl) in the external solution with 50% (w/v) PEG 35K.

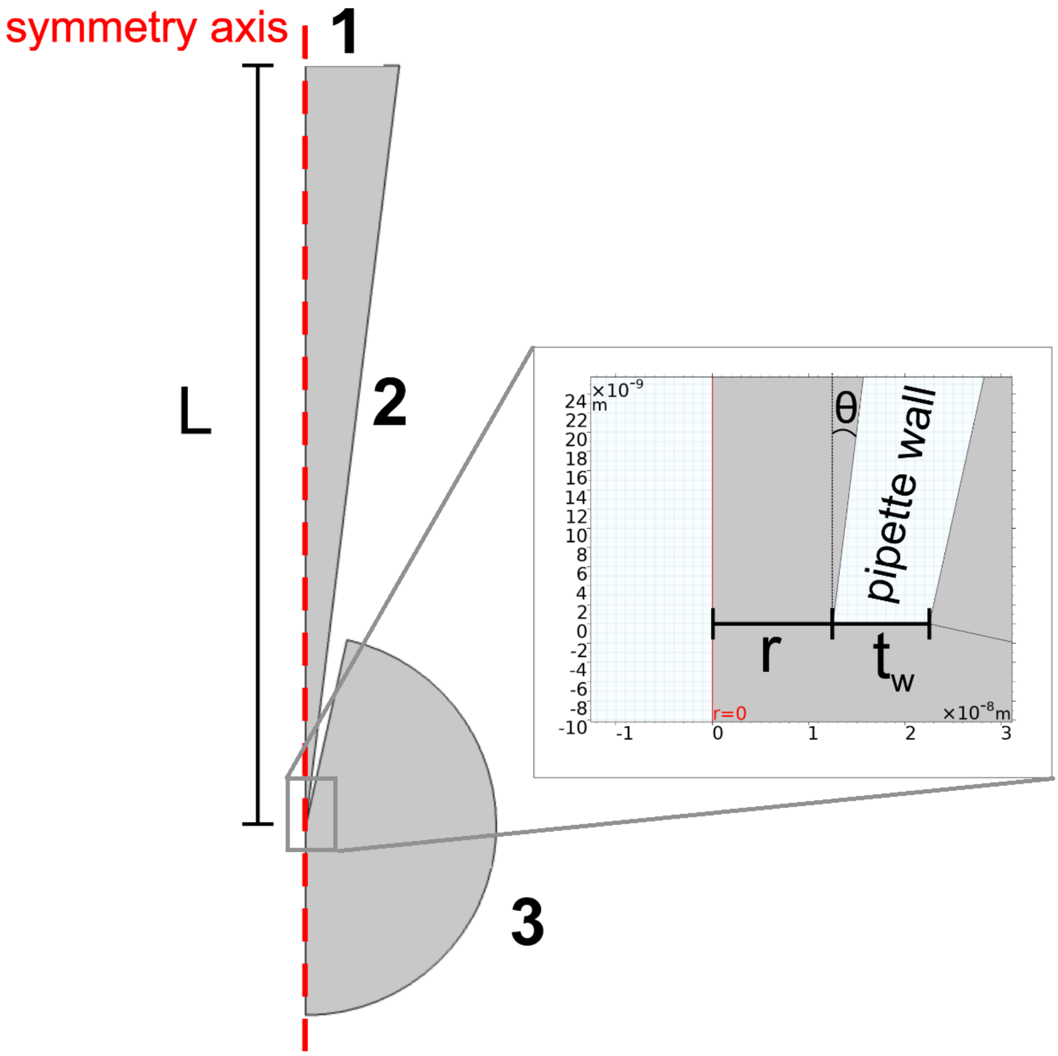

Figure SF1.1 (Left) 2D axisymmetric finite-element model geometry of a simplified nanopipette (truncated cone) immersed in an electrolyte solution, including the symmetry axis (red dashed lines), important boundaries (1, 2, 3) and pore length (L). (Right) Zoomed-in view of nanopipette tip geometry with the pore tip radius (r), inner half-cone angle (θ) and quartz wall thickness (t_w) labelled.

S1.3 Boundary conditions and key numerical parameters

Tables ST1.1 and ST1.2 include the main boundary conditions for the equations explained above and important parameters for the numerical simulations obtained either experimentally or analytically

Table ST1.1 Key boundary conditions applied in finite-element model (no PEG and PEG)

Boundary	Nernst-Planck	Poisson
Bulk solution (1)	$c = c_b = 100\ mM$	$V = V_{app}$
Nanopipette walls (2)	$\hat{n} \cdot \vec{J_i} = 0$	$\sigma = \hat{n} \cdot \epsilon \nabla V$
Bath solution (3)	$c = c_{bath} = 100\ mM$	$V = 0$

Table ST1.2 Key numerical parameters applied in finite-element model (no PEG and PEG)

Parameter	No PEG	PEG	Type
D_{K^+} (m^2/s)	1.7×10^{-9}	1.4×10^{-10}	*best fit to model*
D_{Cl^-} (m^2/s)	1.8×10^{-9}	2.6×10^{-10}	*best fit to model*
κ (S/m)	1.299	0.151	*experimental*
η (Pa·s)	9×10^{-4}	8.73	*experimental (S3)*

S1.4 Mesh

Figure SF1.2 shows the final mesh, which contains 25,684 elements with an average mesh element quality of 81.4%. Further details regarding all the steps required to design the geometry of this model, the boundary conditions and mesh are provided in the COMSOL report present in the data repository (https://doi.org/10.5518/1274).

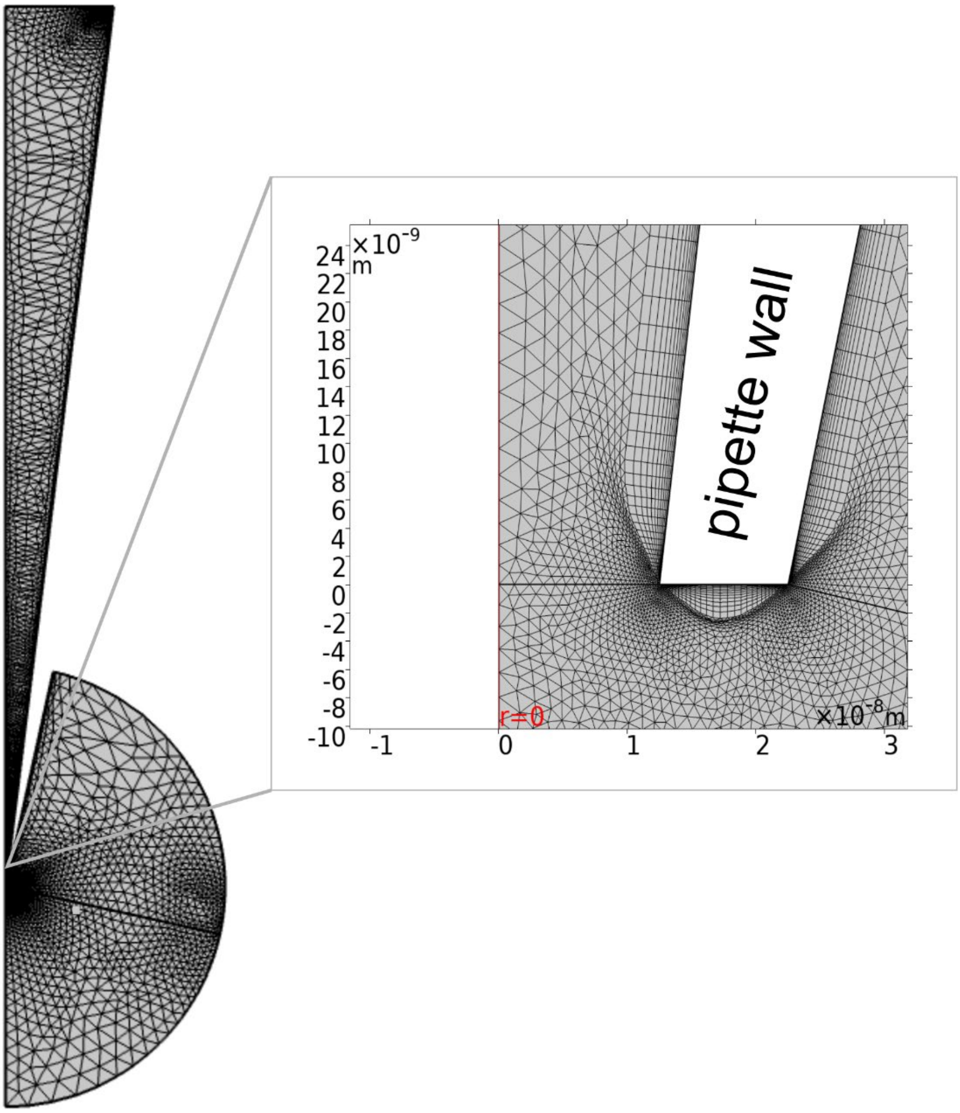

Figure SF1.2: (Left) Final mesh plotted on 2D axisymmetric nanopipette geometry. (Right) Zoomed-in view of mesh at the nanopipette tip aperture.

S1.5 Ion current measurement

The ion current is measured by integrating the ion flux along the top boundary 1 (inner electrode, see Figure SF1.1) according to the following equation:

$$i = 2\pi rF\left(\vec{J}_{K^+} - \vec{J}_{Cl^-}\right) \cdot \hat{n} \qquad \text{(SE1.3)},$$

where $\vec{J}_i$ is the flux of the ion species *i* along the boundary 1 in Figure SF1.1, *F* the Faraday constant and *r* the radial coordinate. In COMSOL, the current probe is added to the definition panel. For more details, see COMSOL report in the data repository (https://doi.org/10.5518/1274).

S2 Definition of simulation input parameters

S2.1 Nanopipette pore diameter

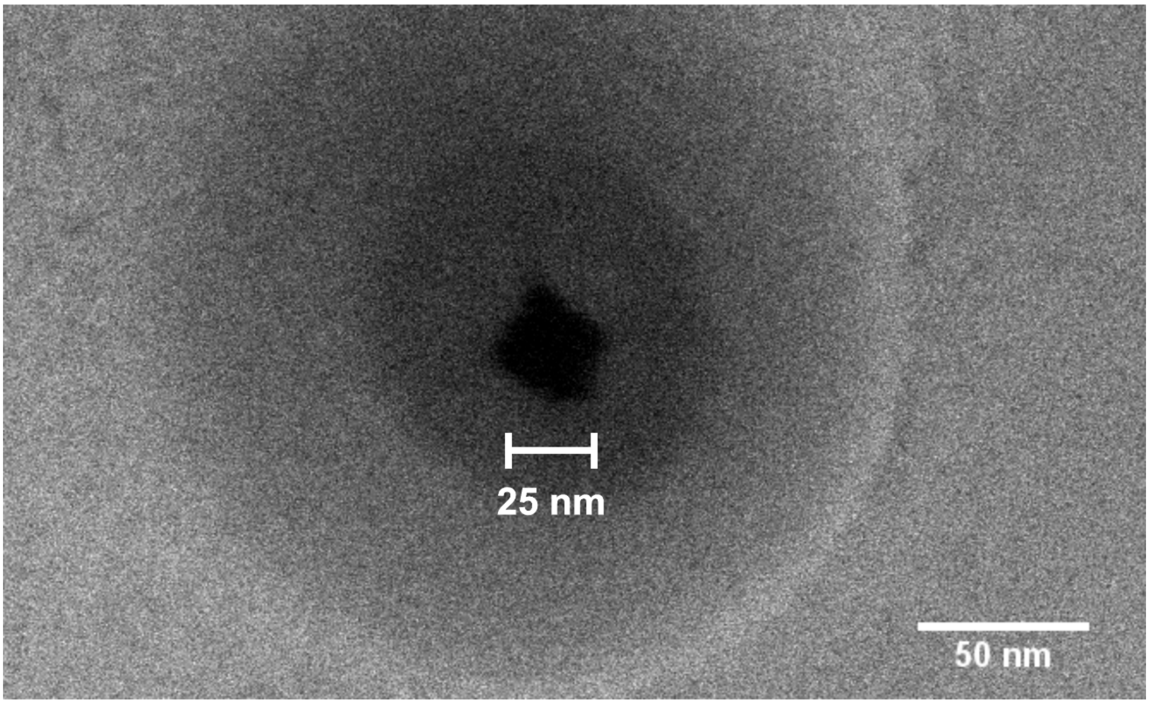

Figure SF2.1: Scanning electron micrograph of the nanopipette used in this work. Image obtained by scanning electron microscopy (Leo 1530 FEG-SEM, Zeiss). Nanopipettes were sputter coated with a gold layer of a few nanometres in thickness. Imaging was performed at 2-3 kV at a working distance of 5 mm.

S2.2 Analytical determination of inner half cone angle

The inner half cone angle was calculated by comparing the measured conductance to an analytical expression for the resistance of the nanopipette, as described below. The resistance of a conical nanopore filled with and immersed in the same electrolyte, which depends on the tip aperture radius (*r*), length of the pore (*L*), electrical conductivity of solution (*κ*) and inner half-cone angle (*θ*), is described by the following equation:

$$R_p = \frac{L}{\pi\kappa r(r + L\tan(\theta))} + \frac{1}{4\kappa r} \qquad \text{(SE2.1)},$$

which for $L\tan(\theta) \gg r$, becomes [2]:

$$R_p = \frac{1}{\kappa r}\left(\frac{1}{\pi\tan(\theta)} + \frac{1}{4}\right) \qquad \text{(SE2.2)}.$$

A patch-clamp amplifier (MultiClamp 700B, Molecular Devices) was used to measure the resistance of a nanopipette (r = 12.5 nm), filled with and immersed in a solution of 0.1 M KCl (κ = 1.299 S/m), which was equal to 175 MΩ. The nanopipette pore radius, r, was measured by scanning electron microscopy (Figure SF2.1) while a conductivity meter was used to measure conductivity, κ. By inputting these values in Equation SE2.2 and solving based on θ, the inner half-cone angle of the nanopipette model is approximately 7°. It is worth noting that Equation SE2.1 provides the same result for the conical pore (L = 50 μm) designed in the model. The resulting simulated i-V curve obtained for the conical geometry described above, but with considering an insignificant (to aid simulation convergence) surface charge on the wall boundaries ($\sigma = 10^{-6}$ C/m$^2 \approx 0$), is ohmic with a slope corresponding to 175 MΩ, as shown in Figure SF2.2. The fact that there is no difference between the simulated and analytically solved resistance of this system, is the first step towards validating the finite-element simulation we developed in this work.

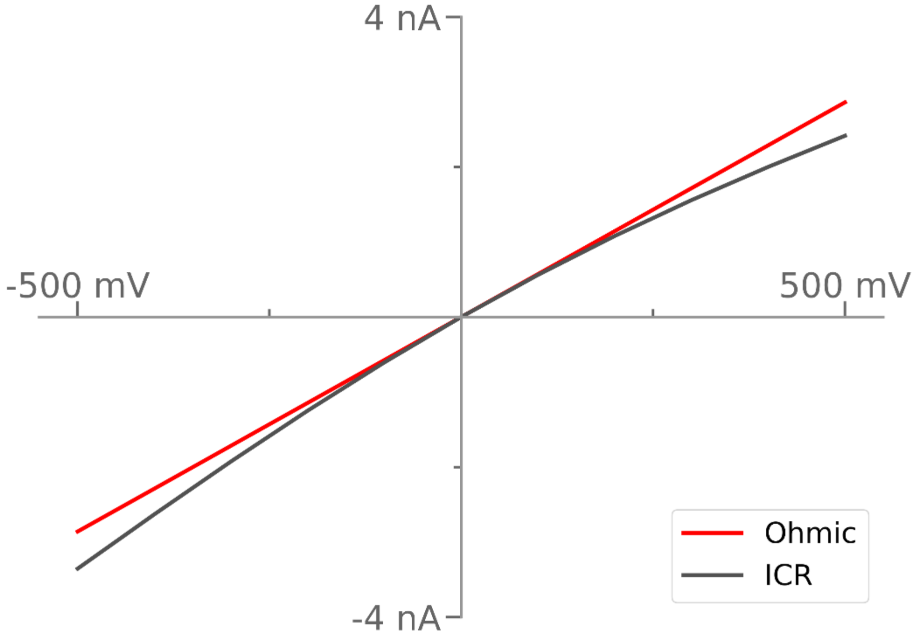

Figure SF2.2 Simulated voltammograms in 0.1M KCl, verifying ohmic *i*-*V* response (red) when no surface charge (σ = 0 mC/m^2) is applied on the nanopipette walls and negative ion current rectification (ICR, black) when σ = -12 mC/m^2.

S2.3 Determination of ion diffusion coefficients

In any electrolyte solution, the ion flux generated by electromigration $\left(\vec{J}_i^m\right)$ is described by:

$$\vec{J}_i^m = -\frac{z_i F}{RT} D_i c_i \vec{E} \qquad \text{(SE2.3)},$$

where $\vec{E}$**:** the electric field[4]. In our system, where $c_b = 0.1$ M KCl, the total electrophoretic flux is equal to the sum of fluxes contributed by the cations $\left(\vec{J}_{K^+}^m\right)$ and anions $\left(\vec{J}_{Cl^-}^m\right)$. This gives the Nernst-Einstein equation, which defines the solution conductivity (κ)

$$\kappa = \frac{(D_{K^+} + D_{Cl^-})}{RT} F^2 c_b \qquad \text{(SE2.4)}.$$

In dilute aqueous solutions, the bulk diffusion coefficients of K^+ and Cl^-, are similar with values of $D_{K^+} = 1.957 \times 10^{-9} \left(\frac{m^2}{s}\right)$ and $D_{Cl^-} = 2.032 \times 10^{-9} \left(\frac{m^2}{s}\right)$, respectively[5]. By adding these two values, we found that potassium cations contribute 49% to the total conductivity while chloride anions contribute the remaining 51%. Based on this and Equation SE2.4, we defined the diffusion coefficient for each ion species through the experimentally measured conductivity $\left(\kappa_{PEG} = 0.15\frac{S}{m}, \kappa_{no\ PEG} = 1.3\frac{S}{m}\right)$ as follows:

$$D_{K^+} = 0.49 \frac{RT}{F^2 c_b} \kappa \qquad \text{(SE2.5)}$$

$$D_{Cl^-} = 0.51 \frac{RT}{F^2 c_b} \kappa \qquad \text{(SE2.6)}$$

When PEG is added to the external bath, this contribution of each ionic species to the total conductivity cannot explain the anomalous *i-V* response. We therefore considered the published evidence of the cation-binding properties of PEG [6]. PEG association with cations in solution was defined by considering an imbalance between the diffusion coefficients of the two species $\left(\frac{D_{K^+}}{D_{Cl^-}} < 1\right)$. A parametric study was performed by increasing the chloride contribution and decreasing the potassium contribution to the total conductivity until the best fit to the

experimental *i*-*V* was obtained (Figure SF2.3). The best fit was found for $\frac{D_{K^+}}{D_{Cl^-}} = 0.54$, meaning that the contribution of potassium to the total conductivity in the presence of PEG is 35% while chloride contributes to the remaining 65%, according to the following equations:

$$D_{K^+}^{PEG} = 0.35\frac{RT}{F^2 c_b}\kappa_{PEG} \quad \text{(SE2.7)}$$

$$D_{Cl^-}^{PEG} = 0.65\frac{RT}{F^2 c_b}\kappa_{PEG} \quad \text{(SE2.8)}$$

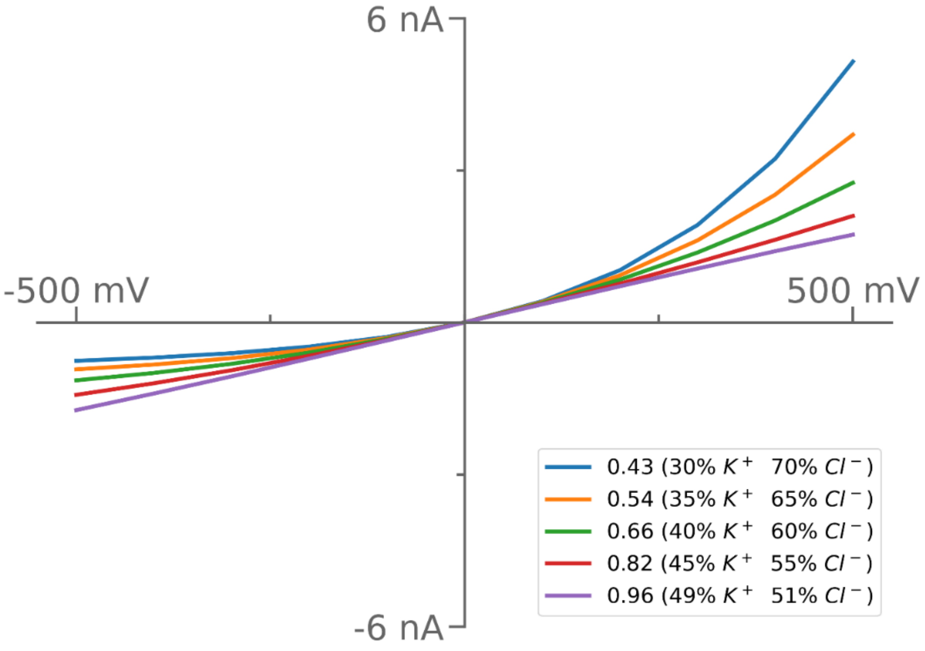

Figure SF2.3 Simulated voltammograms for different ratios between the diffusion coefficient of K^+ and Cl^- in PEG. The orange curve (0.54) was found to be the best fit to the experimental *i*-*V* curve for 0.1 M KCl and 50% (w/v) PEG 35K.

S2.4 Determination of the surface charge on the nanopipette walls

We fit the experimental *i-V* data obtained in 0.1 M KCl (Figure 1b, main text), by adding only a negative surface charge to the boundaries representing the quartz nanopipette walls. Values of surface charge found in the literature[2,3] range from -8 to -24 mC/m^2 and were simulated. Figure SF2.4 illustrates the simulated *i-V* curves for all these values with σ = -12 mC/m^2 providing the closest match to the experimentally measured *i-V* curve.

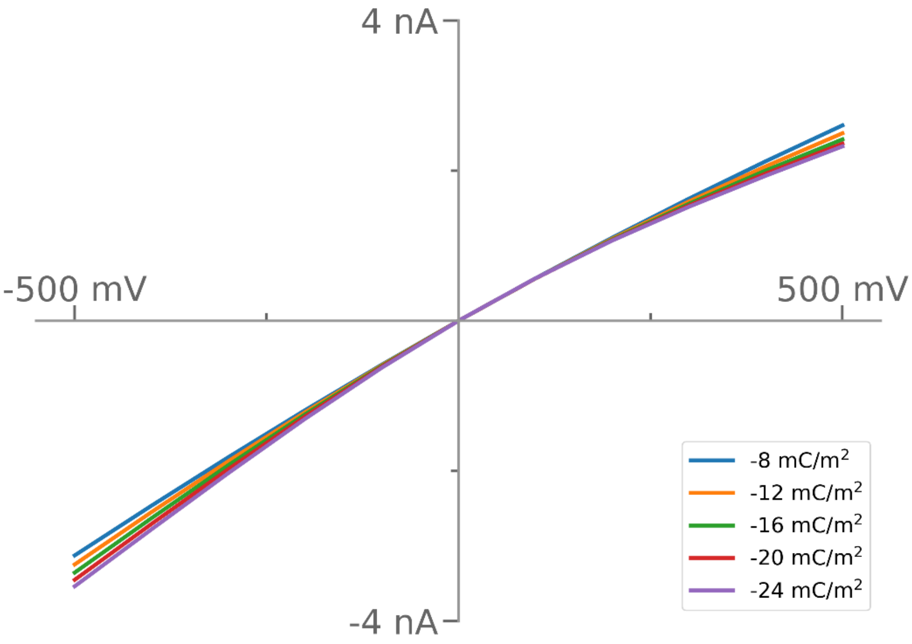

Figure SF2.4 Simulated voltammograms when no PEG is added to the external solution, for a range of applied surface charges on the nanopipette walls. The value -12 mC/m^2 (orange) provided the closest match to the experimental *i-V*.

S2.5 Evidence of negligible influence of surface charge and fluid flow

Once we obtained all the physical parameters required for the finite-element model, we investigated how the surface charge applied on the nanopipette wall boundaries and any fluid flow (*spf*) in the system affected the solution of the simulation (inclusion of the fluid dynamics followed the description given in reference 1; for further details see the Comsol model reports uploaded as additional Supporting Information files). Table ST2.1 presents, after solving the model by activating either the surface charge, the laminar flow, both or none, there were no significant changes in the calculated current for the applied voltage range [-500 mV, +500 mV]. As a result, we decided to deactivate any surface charge on the nanopipette wall boundaries or laminar flow in the system to simplify the simulation solution and illustrate better the concentration of all ion species.

Table ST2.1 Simulated currents for the finite-element model with PEG in the external solution for a voltage range from -500 mV to 500 mV when a combination of the surface charge applied on the nanopipette wall boundaries and any laminar flow in the system are activated/deactivated.

V (mV)	i (nA)			
	$\sigma = 0\ mC/m^2$ No laminar flow	$\sigma = 0\ mC/m^2$ Laminar flow	$\sigma = -12\ mC/m^2$ No laminar flow	$\sigma = -12\ mC/m^2$ Laminar flow
**-500**	-0.93	-0.93	-0.99	-0.99
**-400**	-0.93	-0.83	-0.88	-0.88
**-300**	-0.70	-0.70	-0.73	-0.73
**-200**	-0.53	-0.53	-0.55	-0.55
**-100**	-0.31	-0.30	-0.31	-0.31
**0**	0	0	0	0
**100**	0.40	0.40	0.40	0.40
**200**	0.93	0.93	0.92	0.92
**300**	1.62	1.62	1.60	1.60
**400**	2.52	2.52	2.47	2.48
**500**	3.70	3.71	3.63	3.64

S3 Influence of external solution viscosity in the voltammogram

S3.1 Experimental voltammogram with 50% glycerol in the external solution

To investigate whether voltammograms in PEG can be replicated using a different viscous solution, we experimentally tested the *i-V* responses of a nanopipette filled with 0.1 M KCl when immersed in three different solutions composed of 0.1 M KCl (0.9 mPa·s), 0.1 M KCl with 50% (w/v) PEG 35K (8.73 Pa·s) and 0.1 M KCl with 50% (v/v) glycerol (8.75 mPa·s). Figure SF3.1 proves that the PEG-related *i-V* could not be achieved by adding glycerol in the external 0.1 M KCl solution.

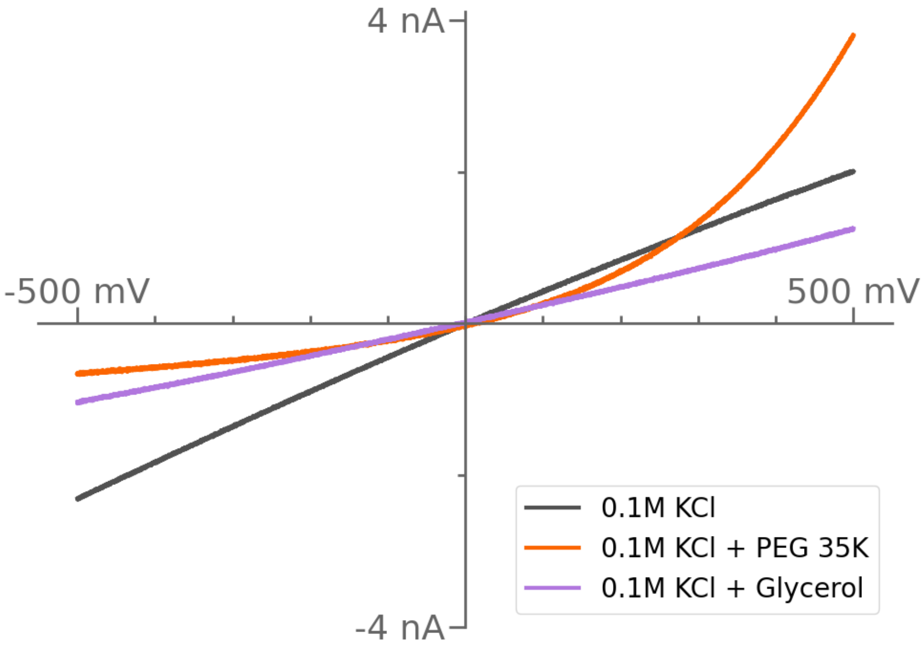

Figure SF3.1 Experimental voltammograms of a nanopipette filled with 0.1 M KCl and immersed in either 0.1 M KCl (gray), 0.1 M KCl with 50% (w/v) PEG 35K (orange) and 0.1 M KCl with 50% (v/v) glycerol (pink).

S3.2 Experimental conductivity and viscosity measurements

Viscosity measurements were carried out using the Kinexus Ultra+ Rheometer (shear rate $1\ s^{-1}$, temperature 25 °C, 5 min acquisition time per sample and recorded data every 30 s, gap between upper and lower geometries 0.15 mm). The geometry used was a cone CP4/40 (4° angle, 40 mm diameter) and plate (60 mm diameter).

Conductivity measurements were carried out using Fisherbrand™ Traceable™ Conductivity Meter Pen (Cat. No. 15-078-200).

S4 Reproducibility of experimental data

S4.1 Voltammograms with three different nanopipettes in the presence of PEG

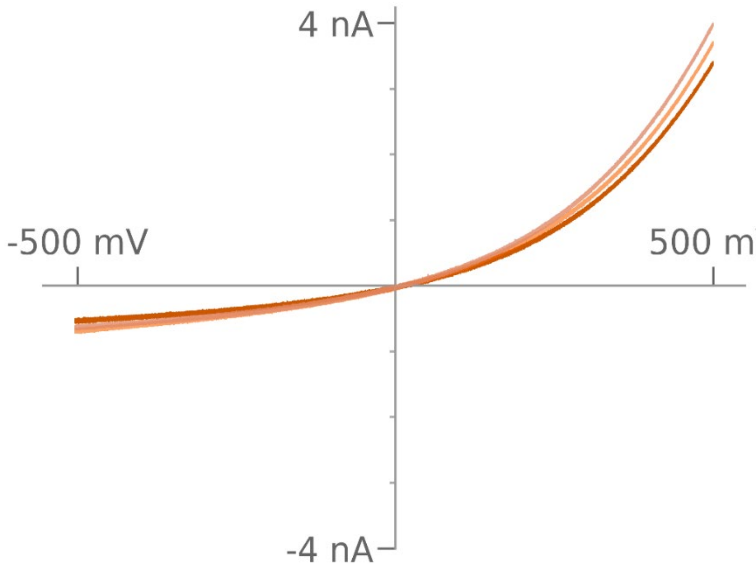

Figure SF4.1: Experimental voltammograms with three different nanopipettes.

S4.2 Voltammograms with three different nanopipettes in absence of PEG

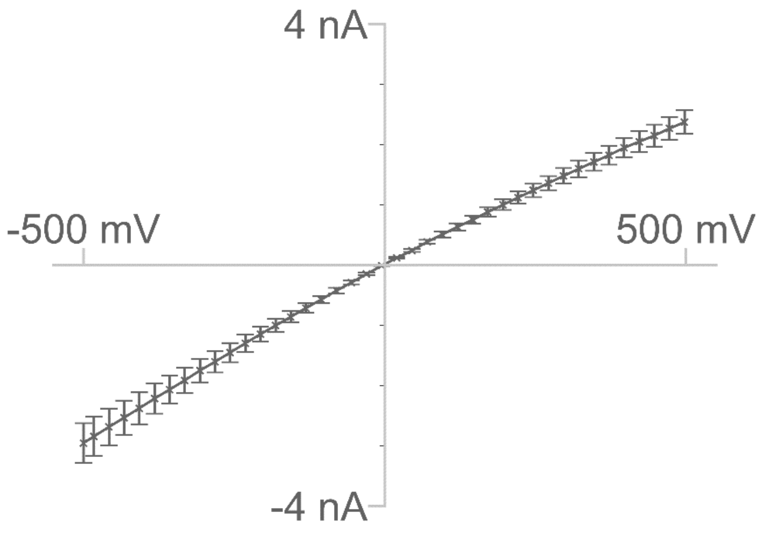

Figure SF4.2: Experimental voltammograms recorded with six different nanopipettes in 0.1M KCl. Error bars correspond to the standard error of the mean.

S4.3 Translocation current recordings with three different nanopipettes

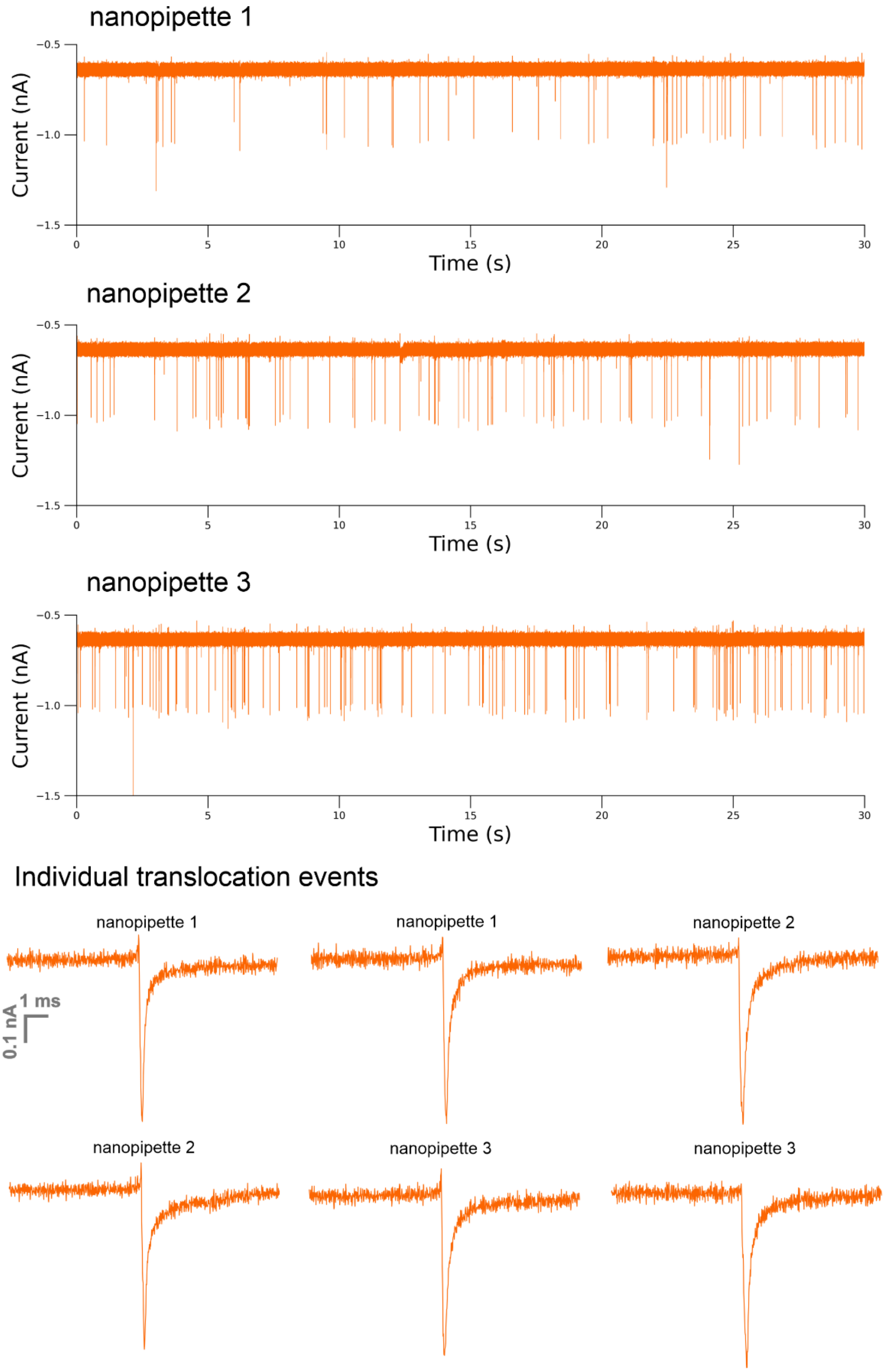

Figure SF4.3: (Top) Translocation current recordings using three different nanopipettes at -500 mV. (Bottom) Individual translocation current events randomly selected from the previous current recordings. For the recordings, the solution inside the nanopipette was 0.3 nM 4.8 kbp dsDNA in 0.1 M KCl while the external solution was 0.1 M KCl + 50% (w/v) PEG 35K.

S4.4 Signal-to-noise (SNR) ratio with and without PEG in the external solution

We measured the noise in terms of root mean square value (RMS) of the baseline current from three traces recorded in the presence of PEG in the external solution and three traces in absence of PEG (all measured at -500 mV). We obtained the following values:

$$I_{PEG}^{RMS} = 15.1 \pm 1.6 \text{ pA}$$

$$I_{no\ PEG}^{RMS} = 12.8 \ \pm 0.3 \text{ pA}$$

The SNR values $\left(SNR = \frac{|\Delta I|}{I_{RMS}}\right)$ for each event detected (current traces in Figure SF4.4a) using the same nanopipette upon translocation of 4.8 kbp dsDNA molecules (our sample analyte) with (orange) and without (gray) PEG are illustrated in Figure SF4.3b.

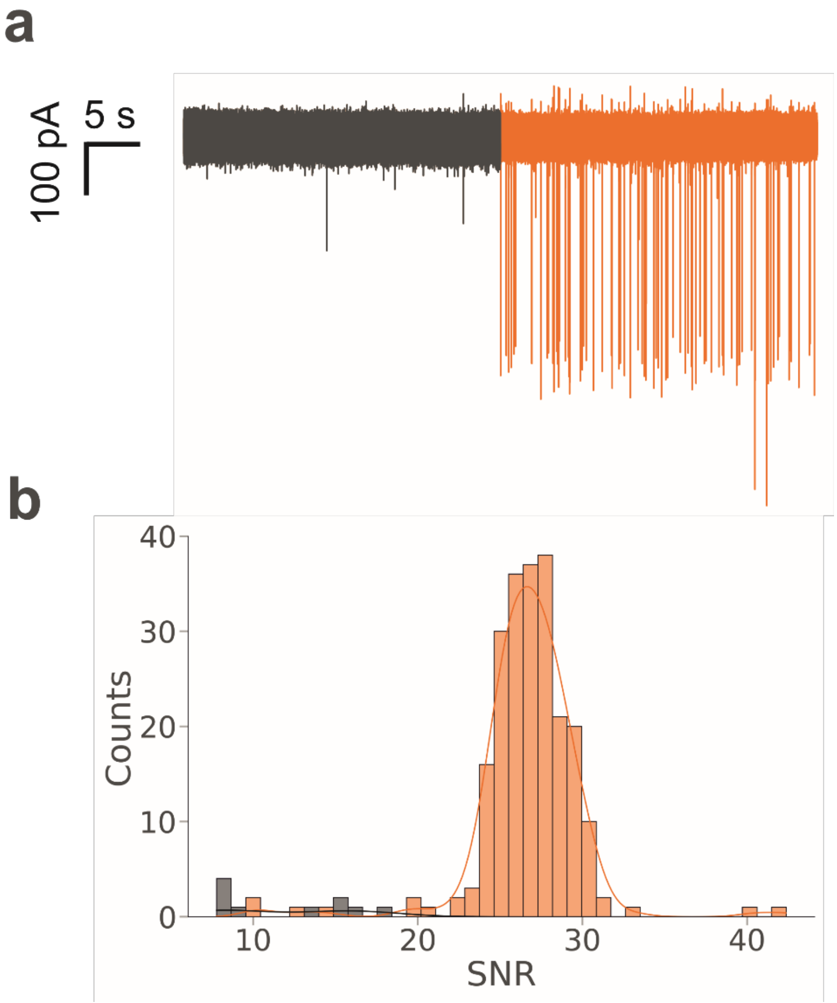

Figure SF4.4: (a) Ion current traces using the same nanopipette showing the translocation of 4.8 kbp dsDNA molecules in the absence (gray) and presence (orange) of PEG in the external solution. (b) Histogram showing the SNR calculated for each event of the ion current traces (a) in the absence (gray) and presence (orange) of PEG in the external solution. Measurements performed at -500 mV applied potential.

S4.5 Current recordings with no analyte in the nanopipette with PEG in the external solution

To check whether the conductive events were due to the PEG molecules translocating through the nanopipette we repeated the same experiment showed in Figure 1 (main manuscript) where no analyte was added to the nanopipette solutions. All current recordings showed no translocation events, suggesting that PEG molecules do not translocate through the pore and that the signal is uniquely due to the translocation of dsDNA molecules.

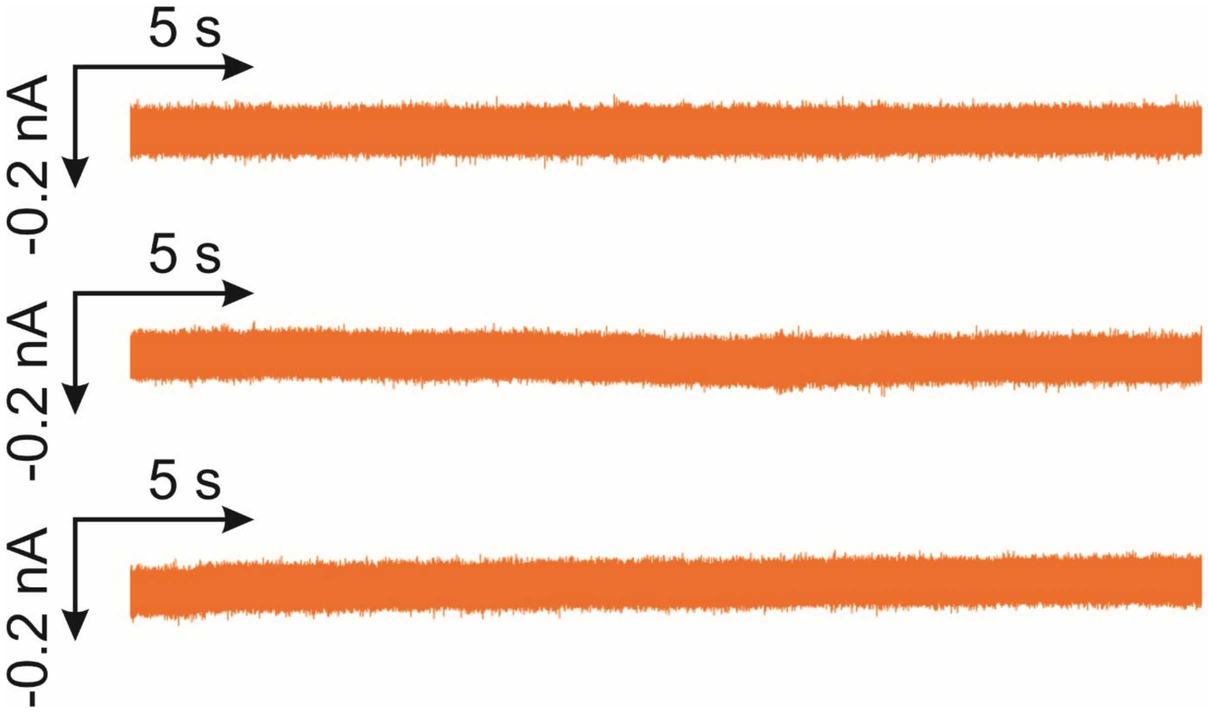

Figure SF4.5: Current traces recorded with three different nanopipettes where no analyte was added to the nanopipette solution. The nanopipettes were filled with 0.1 M KCl and immersed in a 0.1 M KCl bath containing 50% (w/v) PEG 35K. A voltage bias of -500 mV was applied.

S4.6 Continuous measurement of dsDNA translocation into the PEG solution

We performed a 20-minute continuous measurement of dsDNA translocation to check if the presence of PEG changes the properties of translocation events over time. Within our longest experiment duration we performed, our analysis shows that there are no significant changes in both the number of translocation events (Figure SF4.6) and the translocation peak characteristics (Figure SF4.7) in the first 2 mins (from 0 to 20 mins) versus the last 2 mins (from 18 to 20 mins).

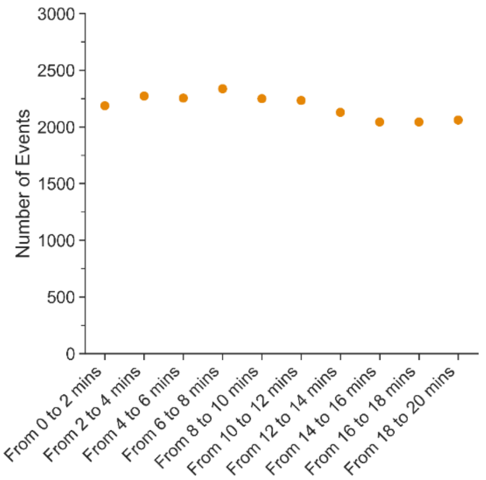

Figure SF4.6: 4.8 kbp dsDNA were translocated from the nanopipette to the 50% PEG KCl bath for a duration of 20 mins. The translocation events were analysed every 2 mins. The average number of the translocation events detected is 2181 ± 33 (Standard Error of the Mean).

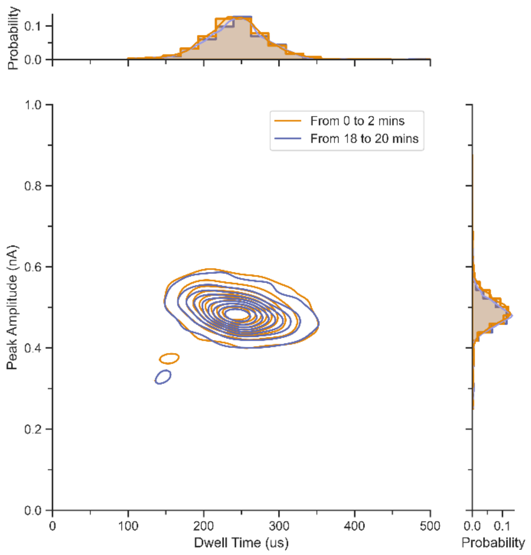

Figure SF4.7: Kernel density plot (KDE) of the events from 0 to 2 minutes and from 18 to 20 minutes overlapped in terms of Current Amplitude (pA) and Dwell Time (μs). No significant difference was observed.

S4.7 Translocation traces over an extended period.

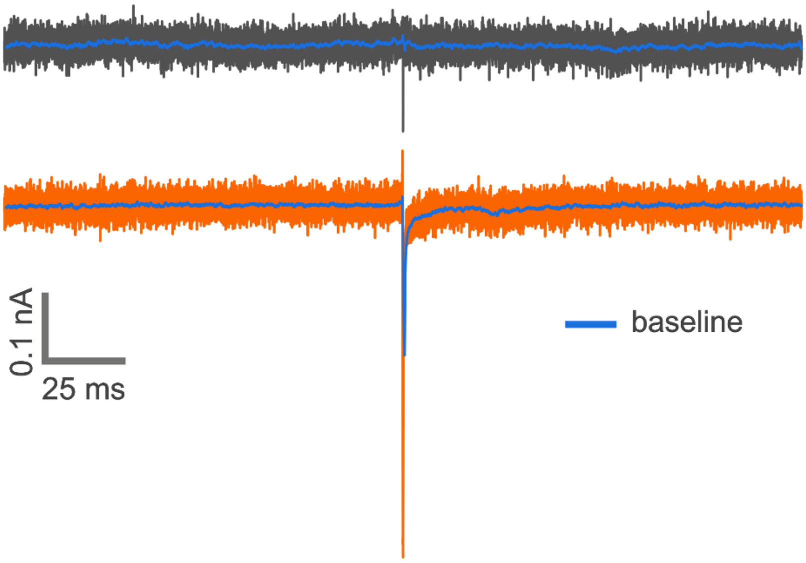

Figure SF4.8: Current traces show in Figure 1c (main manuscript) over an extended period of time. The full length of the traces is 200 ms. The baseline level of the signals was calculated with a rolling average with window length equal to 0.5 ms.

S5 Ion concentrations at the nanopipette tip region

S5.1 Individual cation and anion concentrations along the symmetry axis

Ion concentrations are plotted in terms of average concentrations $C_{avg} = \frac{[K^+]+[Cl^-]}{2}$ in the main text, due to the very similar cation and anion distributions in the entire model. Figure SF5.1 illustrates the cation (K^+) and anion (Cl^-) concentrations along a portion of the axis of symmetry (red dashed line, Figure SF1.1) when PEG is present (orange) or absent (black) in the external solution under an applied voltage of -500 mV (dashed line) and +500 mV (solid line). Both ion concentrations tend to reach the value of the initially applied concentration (100 mM) for z < -200 nm and z > 600 nm.

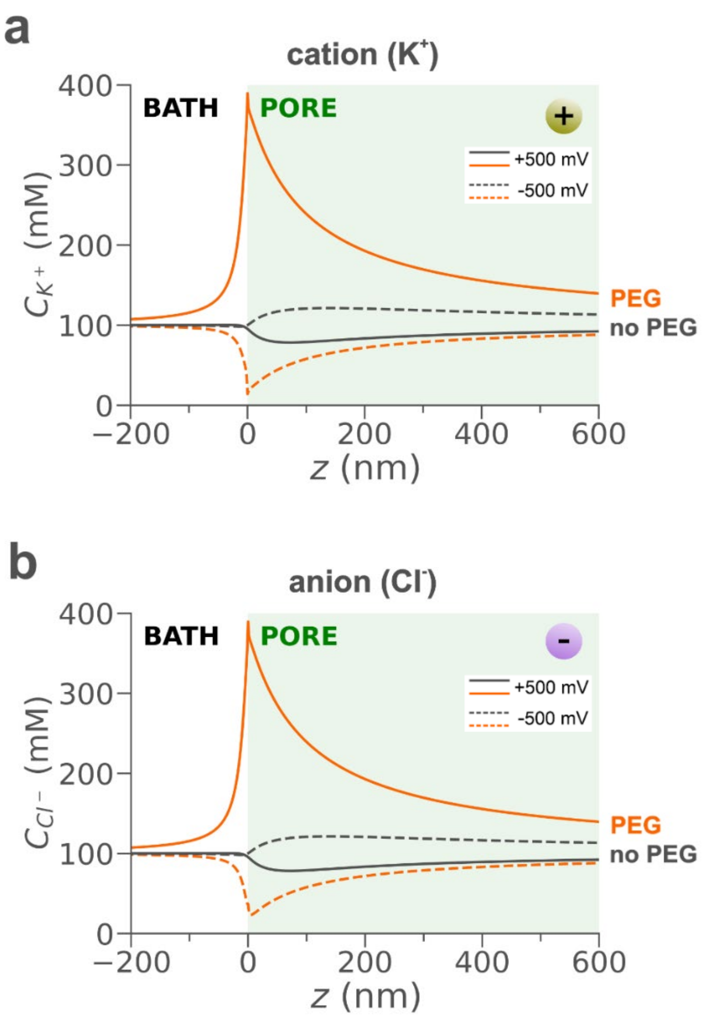

Figure SF5.1: a) Cation (K^+) and b) anion (Cl^-) concentrations along 800 nm of the nanopipette axis of symmetry (red dashed line in Figure SF1.1) in presence (orange) and absence (black) of PEG for -500 mV (dashed curves) and +500 mV (solid curves). The diameter of the nanopipette is 25 nm and the internal and external solution is 0.1 M KCl for both PEG and no PEG, but in the PEG case, the external solution also contains PEG 35K.

S5.2 Average ion concentrations along the symmetry axis under different applied potentials for the PEG condition

Figure 2a, b in the main text focus only on the average ion concentration along the nanopipette symmetry axis when -500 mV and +500 mV are applied, respectively, and PEG is present in the external solution. Figure SF5.2 presents how the average ion concentration C_{avg} is influenced when the applied potential is changed from -500 mV to +500 mV with a 100 mV step. It is evident that, as the positive applied potential becomes lower, ion enrichment close to the interface (z = 0 nm) is reduced, while as the negative applied potential becomes larger, the average ion concentration at z = 0 nm increases.

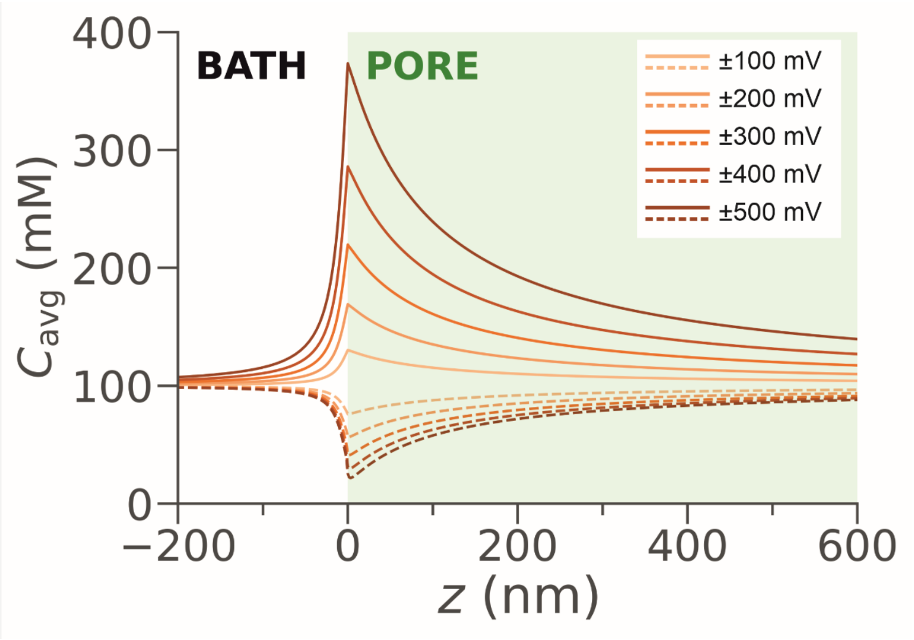

Figure SF5.2: Average ion concentrations along the nanopipette axis of symmetry in the presence of PEG under different applied potentials [-500, +500 mV].

S6 Ion transport at the tip region and definition of sensing region

S6.1 Boundaries for the calculation of ion transport

The geometry design analyzed in SF1.1 and in the COMSOL report in the data repository (https://doi.org/10.5518/1274) was slightly modified by adding semi-circular and semi-elliptical boundaries around the nanopipette tip opening which could then be used as curves to calculate the integral of the normal ion fluxes, and hence the transport rates of each ion species. Initially, we designed a semi-circle with radius 1.5 μm and center coordinates (0, 0 nm), which was divided into smaller ones by adding 11 layers, as shown in Figure SF6.1a. Then, a semi-ellipse with major semi-axis of 22.5 nm, minor semi-axis of 10 nm and center at (0, 0 nm) was also added in the geometry, which was also split into 10 smaller ones by reducing the minor semi-axis with a step of 1 nm, except the last which had a step size of 0.7 nm (Figure SF6.1b). All these correspond to the simulation where PEG is present in the external solution.

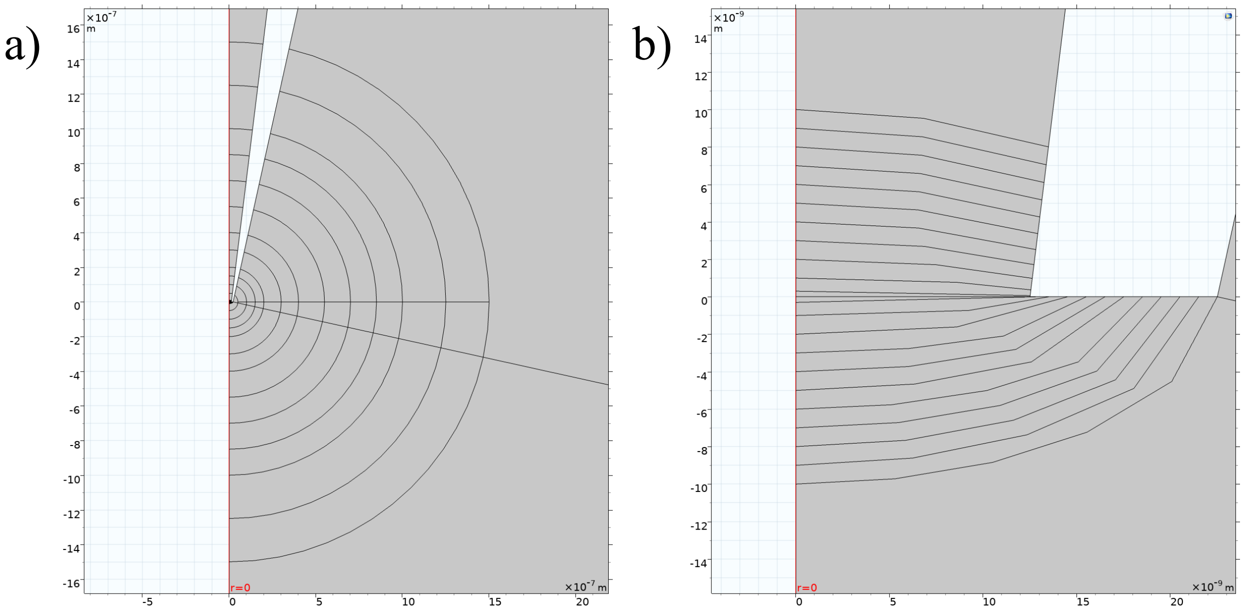

Figure SF6.1 COMSOL zoomed-in geometry plots showing a) semi-circular boundaries inside and outside the nanopipette pore with common centre at (0 nm, 0 nm) and radii ranging from 50 nm to 1.5 μm and b) semi-elliptical boundaries inside and outside the nanopipette pore with common centre at (0 nm, 0 nm) and minor semi-axis ranging from 0.3 nm to 10 nm.

S6.2 Calculating the transport rates of each ion species at the boundaries

As previously explained, since convection (*Laminar Flow*) did not influence these simulations, the total ion flux $(\vec{J}_i)$ is only composed of the electromigrative and diffusive ion fluxes $(\vec{J}_i^m, \vec{J}_i^d)$. Based on Equation SE6.1, the total transport rate of each ion species (N_{K^+}, N_{Cl^-}) was calculated by integrating the normal $\vec{J}_i$, $\vec{J}_i^m$ and $\vec{J}_i^d$ along each designed boundary around the nanopipette tip (Figure SF6.1)[8].

$$N_i = \int_{\partial\Omega} \vec{J}_i \cdot \hat{n} ds = \int_{\partial\Omega} \vec{J}_i^m \cdot \hat{n}\, ds + \int_{\partial\Omega} \vec{J}_i^d \cdot \hat{n} ds = N_i^m + N_i^d \qquad \text{(SE6.1)}$$

where $\partial\Omega$: boundary of integration and *ds* the surface element. Figure SF6.2 shows the total, electrophoretic and diffusive transport rates of cations and anions for positive and negative bias applied.

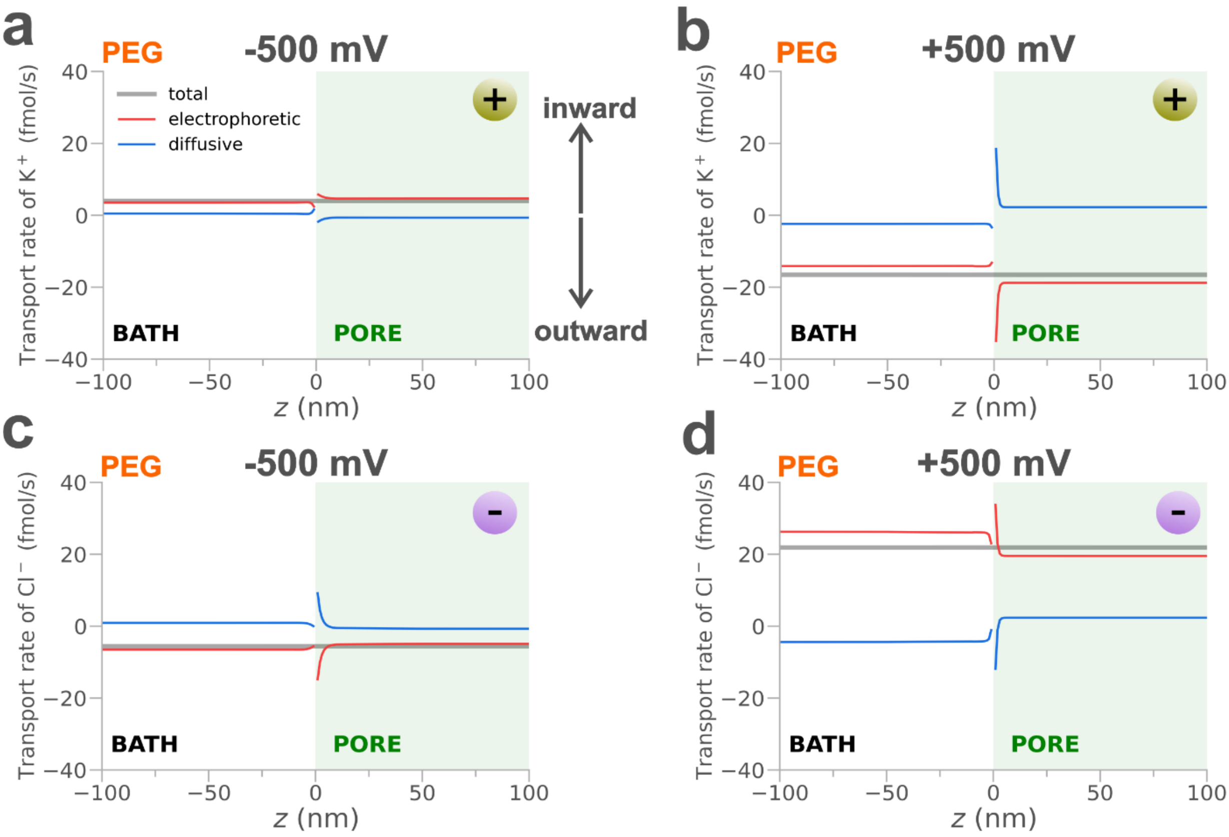

Figure SF6.2 Total, electrophoretic and diffusive transport rates of a) K^+ for -500 mV, b) K^+ for 500 mV, Cl^- for -500 mV and d) Cl^- for -500 mV when PEG is present to the outside bath. The horizontal axis (z) represents the radius in nm of the designed boundaries, as explained in section S6.1. The green positive and purple negative spheres represent K^+ and Cl^-, respectively. The inward/outward arrows represent movement of each ion species towards inside/outside the nanopipette tip opening.

S6.3 Defining the "sensing region" based on the electric potential distribution

Figure SF6.3a illustrates the electric potential distribution around the nanopipette tip region when PEG is added to the external solution. By drawing equipotential lines (each curve represents a constant value for the electric potential in that domain), we determined that 50% of the applied voltage in the model (ΔV_{sens} = 250 mV) drops within approximately ±20 nm from the tip interface. This region (40 nm along the z-axis) was defined as the "sensing region" of this system (dashed lines I and IV, Figure 3).

In addition, Figure SF6.3b shows the distribution of the electric potential (V) along the z-axis of the geometry for the presence (orange curve) and absence (gray curve) of PEG in the outside solution. An inset from z = -200 nm to z = 200 nm was included to depict the difference of the "sensing region" in size between the case of PEG and no PEG. To define the "sensing region" for the latter case, we used the equipotential line at -20 nm as the starting point and found the equipotential line inside the pore (120 nm) that reached the same ΔV as in PEG.

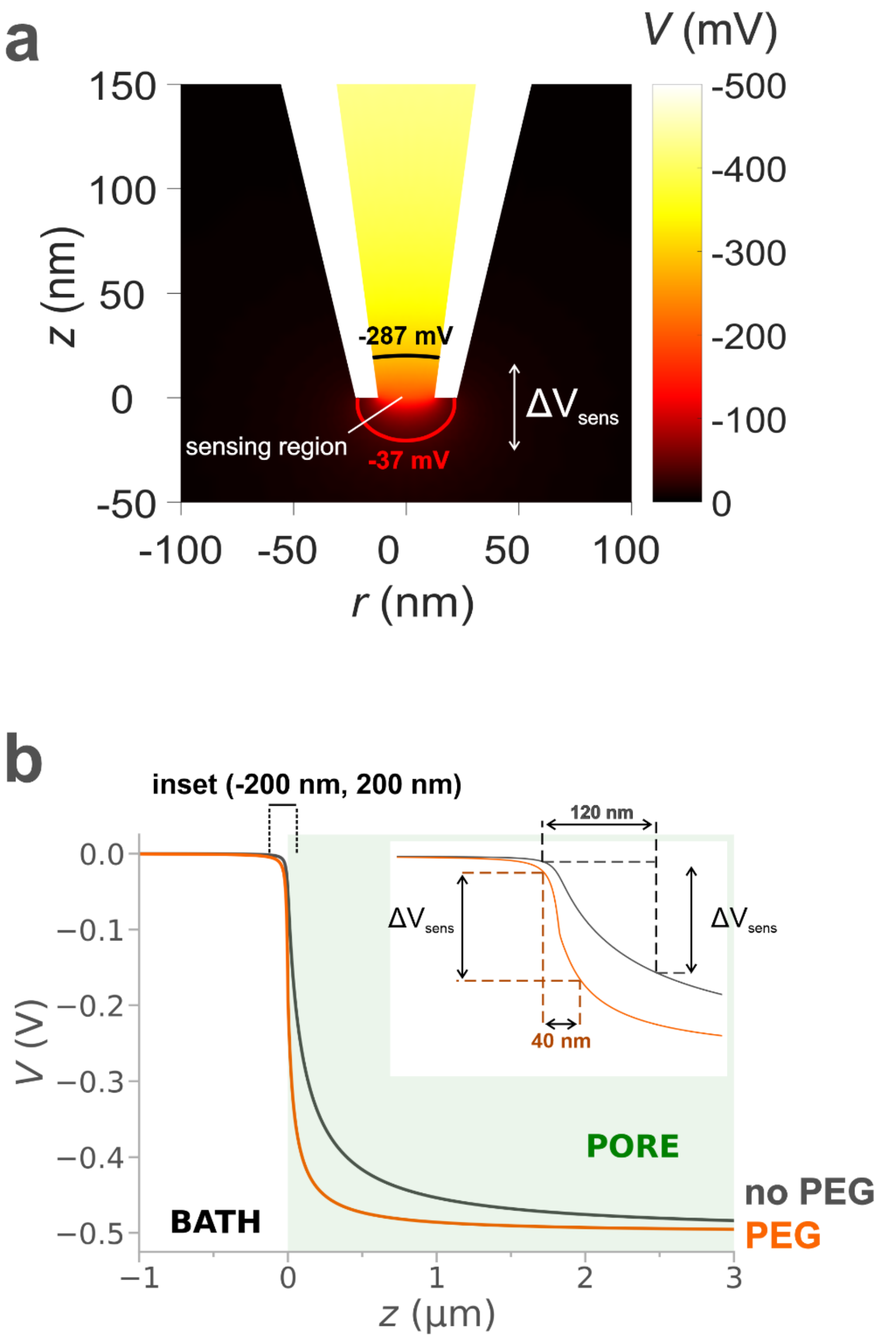

Figure SF6.3 a) Surface colour plot of the electric potential distribution around the nanopipette tip region. The two highlighted curves represent equipotential lines for -287 mV and -37 mV illustrating the "sensing region" where half of the applied voltage drops (ΔV_{sens} = 250 mV) in the presence of PEG. b) Electric potential along the symmetry z-axis of the model geometry (r = 0 nm) for both cases where PEG is present (orange) and absent (gray) from the outside bath. The initial applied voltage is -500 mV at the top boundary of the nanopipette. The inset focuses on the voltage between -200 nm ≤ z ≤ 200 nm to depict the differences between the two "sensing regions" (PEG and no PEG).

S6.4 Transport rates of each ion species in the sensing region

Figure SF6.4 presents the COMSOL geometry of the finite-element model with two additional boundaries that mimic the shape of the equipotential lines for -287 mV and -37 mV at z = ±20 nm, respectively (Figure SF6.3a). The surface area enclosed between these two boundaries represents the "sensing region" when PEG is added in the external solution.

To define the transport rates of each ion species in the "sensing region" of the PEG case, we integrated the normal total, electrophoretic and diffusive ion fluxes for K^+ and Cl^- as explained in section S6.2. The integration was performed along the two boundaries mentioned above (z = ±20 nm) and two additional semi-elliptical boundaries (Figure SF6.1b) at z = ±5 nm.

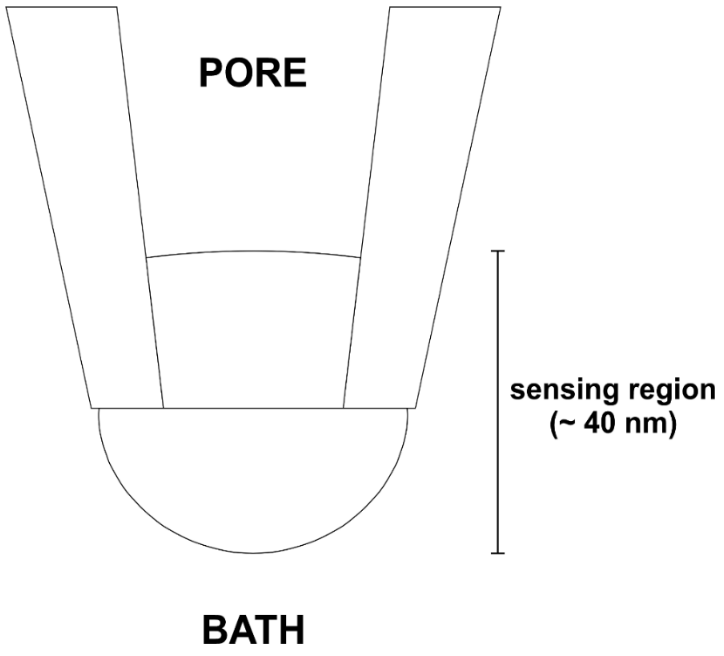

Figure SF6.4 COMSOL geometry design of the finite-element model used to calculate the transport rates of each ion species, with the addition of two boundaries identical to the equipotential lines that represented the "sensing region" when PEG is added in the external solution.

The following tables (ST6.1, ST6.2) present the electrophoretic, diffusive and total transport rate values of potassium cations and chloride anions for ±500 mV with PEG in the external solution. The values at z = ±5 nm, were obtained from the initially designed semi-elliptical boundaries (section S6.1).

Table ST6.1 Electrophoretic (N_i^m), diffusive $\left(N_i^d\right)$ and total (N_i) transport rate of K^+ and Cl^- in [fmol/s] for -500 mV with PEG in the external solution at the height (z) of 4 equipotential boundaries close to the nanopipette tip. Negative/positive values represent directionality outwards/inwards the nanopipette tip opening.

**-500 mV**						
**z [nm]**	$\boldsymbol{N_{K^+}^m}$ **[fmol/s]**	$\boldsymbol{N_{K^+}^d}$ **[fmol/s]**	$\boldsymbol{N_{K^+}}$ **[fmol/s]**	$\boldsymbol{N_{Cl^-}^m}$ **[fmol/s]**	$\boldsymbol{N_{Cl^-}^m}$ **[fmol/s]**	$\boldsymbol{N_{Cl^-}}$ **[fmol/s]**
**-20**	3.53	0.49	4.02	-6.53	0.93	-5.60
**-5**	3.56	0.46	4.02	-6.28	0.68	-5.60
**5**	4.79	-0.77	4.02	-5.60	-0.001	-5.60
**20**	4.68	-0.66	4.02	-4.94	-0.66	-5.60

Table ST6.2 Electrophoretic (N_i^m), diffusive $\left(N_i^d\right)$ and total (N_i) transport rate of K^+ and Cl^- in [fmol/s] for +500 mV with PEG in the external solution at the height (z) of 4 equipotential boundaries close to the nanopipette tip. Negative/positive values represent directionality outwards/inwards the nanopipette tip opening.

**+ 500 mV**						
**z [nm]**	$\boldsymbol{N_{K^+}^m}$ **[fmol/s]**	$\boldsymbol{N_{K^+}^d}$ **[fmol/s]**	$\boldsymbol{N_{K^+}}$ **[fmol/s]**	$\boldsymbol{N_{Cl^-}^m}$ **[fmol/s]**	$\boldsymbol{N_{Cl^-}^m}$ **[fmol/s]**	$\boldsymbol{N_{Cl^-}}$ **[fmol/s]**
**-20**	-14.07	-2.35	-16.42	26.01	-4.27	21.74
**-5**	-14.03	-2.39	-16.42	25.53	-3.79	21.74
**5**	-18.65	2.23	-16.42	19.42	2.32	21.74
**20**	-18.65	2.23	-16.42	19.42	2.32	21.74

S7 Mechanism of current enhancement upon dsDNA translocation

S7.1 Estimating the number of ions carried by single dsDNA in the "sensing region"

So far, we explained the differences in the baseline current magnitude between the PEG and no PEG models by analyzing the ion concentration distributions and transport rates close to the nanopipette tip, especially in the "sensing region". In this section, we attempt to unravel the mechanism behind the enhanced current magnitude when dsDNA molecules translocate through the nanopipette aperture towards the PEG-enriched bath.

At first, we calculated the number of ions inside the sensing region, illustrated in Figure SF6.4, for both cases (PEG, no PEG). Based on Figure SF5.1, the average ion concentration in the sensing region is 52 mM and 102 mM for the PEG and no PEG case, respectively. By surface integration of these values, we obtained the total number of ions for both the PEG and no PEG models as follows:

$$n_t = 2\pi N_A \iint rC_{avg}\, drdz = \pi N_A \iint r(c_{K^+} + c_{Cl^-})drdz \qquad \text{(SE7.1)},$$

where N_A: Avogadro's constant and c_{K^+}, c_{Cl^-}: K^+ and Cl^- concentration, respectively.

We estimate the presence of 972 ions within the sensing reason when PEG is added to the external solution compared to 1895 ions when no PEG is added. These values correspond to an ion current at V = -500 mV of -0.92 nA and -3.27 nA, respectively. When single dsDNA molecules translocate through the sensing region towards the external solution, we observe current peaks of -1.38 nA with PEG and -3.40 nA without PEG. To achieve such values, the number of ions in the sensing region would have to increase by approximately 33% (~320 ions) and 4% (~75 ions), respectively. However, we would expect the number of counterions carried by dsDNA molecules in the sensing region to be the same for both cases. This is an indication of more complex phenomena associated with the translocation of the molecule and the interface between 0.1 M KCl and 0.1 M KCl with PEG.

S7.2 Model for interface displacement due to dsDNA translocation

To address the influence of the displacement of the internal-external solution influence on the current response during the translocation of a dsDNA molecule, we developed a model with a cylindrical plug of internal solution protruding beyond the pipette orifice. The plug had a fixed radius of 12.5 nm (equal to pore tip radius) and a height ranging from 2 nm to 30 nm, for $z < 0$ nm (Figure SF7.1a, b). The boundary conditions allocated to this new domain were identical to those applied in the nanopipette pore domain. Then, we solved the simulation to find which height provided the closest current value to the experimental translocation peak current (Figure 1c, main text).

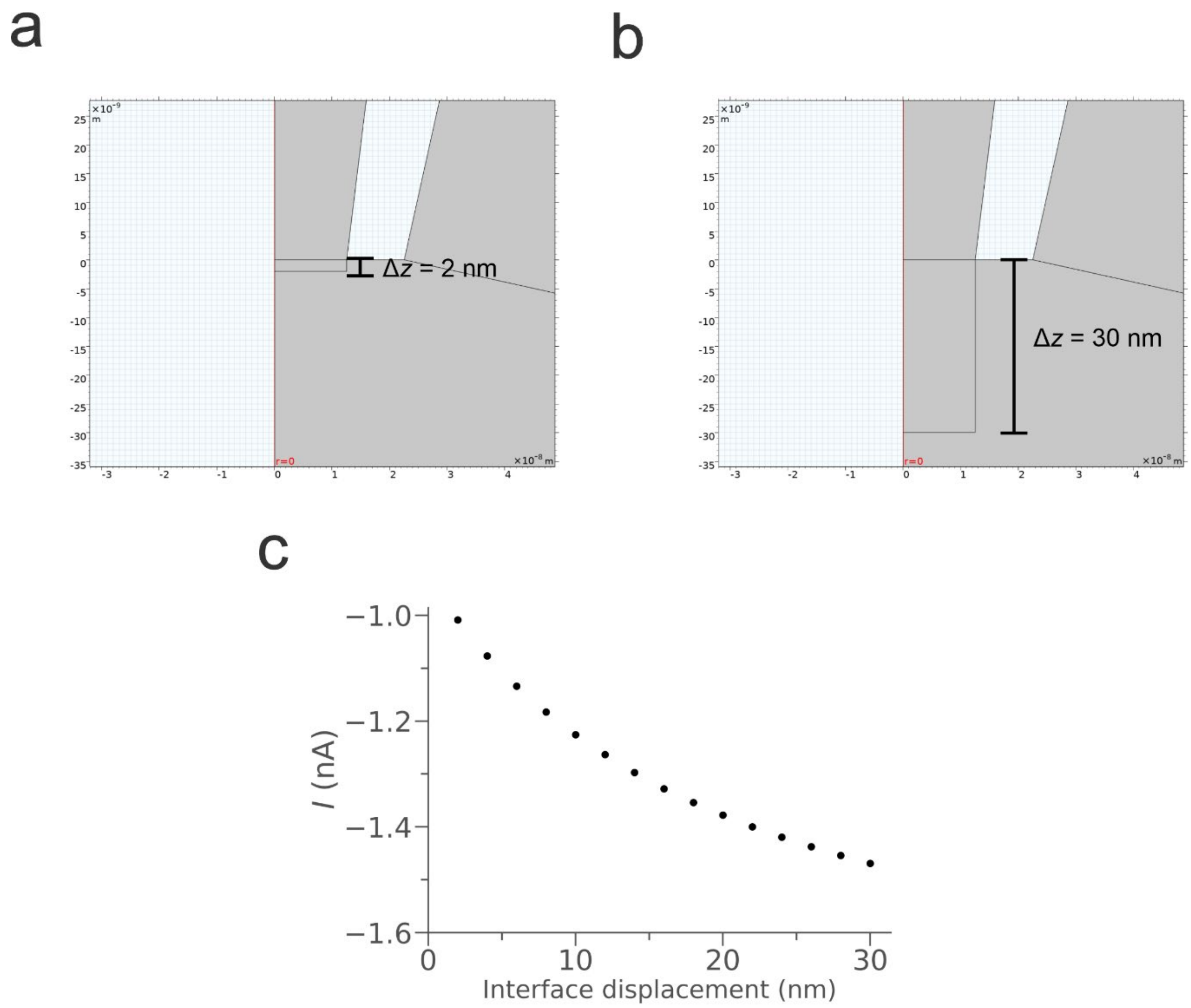

Figure SF7.1 Model for interface displacement towards the bath solution. a, b) Exported COMSOL geometries for the minimum and maximum interface displacement along the z-axis with a fixed width equal to the radius of the nanopipette tip aperture (12.5 nm). c) Simulated current for different interface displacements towards the bath solution.

Depending on the length of the interface displacement, the current peak magnitude increases significantly until 20 nm where it starts to saturate (Figure SF7.1c). At z = -16 nm, the simulated current has a magnitude of -1.33 nA which agrees with the experimental value for a 4.8 kbp dsDNA translocating through the nanopipette (Table ST7.1).

S7.3 Effect of dsDNA size on translocation current

In this section, we investigate the effect of the dsDNA size on the experimentally recorded translocation current. By using the same experimental configuration as shown in Figure 1 in the main text, we recorded a trace of 30 s for every dsDNA molecule size (0.7, 1.5, 2, 3, 4, 4.8 and 7 kbp, agarose gel in Figure SF7.3) where a different nanopipette was used for each size for both PEG and no PEG cases. Figure SF7.2a shows a representative event for each current trace with bigger dsDNA molecules corresponding to higher current peak magnitudes. All events per trace are illustrated in the scatter plot in Figure SF7.2b in terms of current peak maxima and dwell time, where the latter is the duration of the event calculated at full-width half-maximum. The dsDNA size can be easier discriminated using the current peak maxima values compared to their dwell times.

By calculating the mean current peak maxima and standard deviation for each set of events we observed a dependency between the peak maxima and dsDNA molecule size, as depicted in Figure SF7.2c (orange data points). The same trend is not noticeable for the case without PEG in the external solution (gray data points) with a detection limit of 4.8 kbp.

Table ST7.1 Experimental and simulated translocation current magnitudes for each dsDNA size and interface displacement.

**dsDNA size (kbp)**	**0.7**	**1.5**	**2**	**3**	**4**	**4.8**	**7**
**Experimental Current Peak Maxima (nA)**	0.13	0.18	0.24	0.30	0.37	0.41	0.44
**Displacement (nm)**	**4**	**6**	**8**	**10**	**14**	**16**	**18**
**Simulated Current Peak Maxima (nA)**	0.16	0.21	0.26	0.30	0.38	0.41	0.43

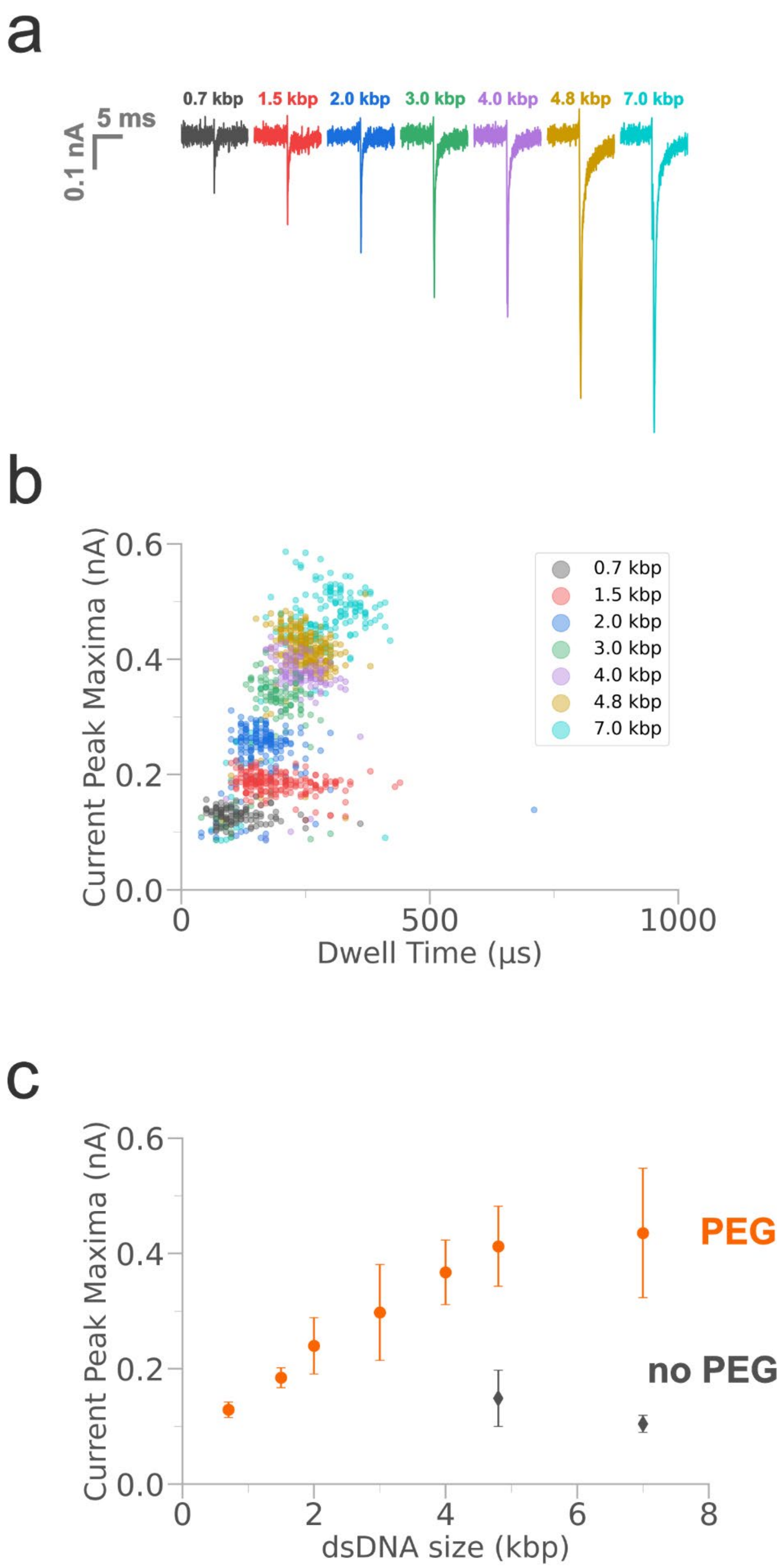

Figure SF7.2 Experimental translocation currents for different dsDNA molecule sizes. a) Representative event for each dsDNA size showing current peaks. b) Scatter plot for all events for each dsDNA size in terms of current peak maxima versus dwell time. c) Mean current peak maxima over size of dsDNA molecules translocating through the nanopipette tip aperture towards the bath with (orange) and without (gray) PEG. The error bars represent the standard deviation.

S7.4 Agarose gel electrophoresis of dsDNA samples

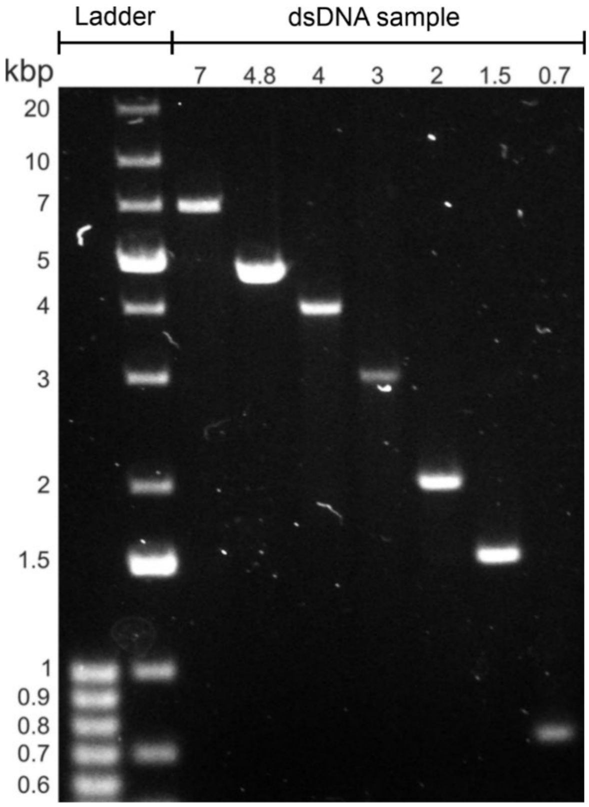

Figure SF7.3: Agarose gel electrophoresis analysis of the dsDNA samples extracted from a 1 kbp plus DNA ladder (SM1331: Thermo Fisher). Samples were run along two dsDNA ladder (GeneRuler 100 bp DNA ladder and GeneRuler 1 kb plus DNA ladder) to allow better discrimination of all sizes.

S7.5 Effect of an externally applied pressure to the nanopipette

The i-V curves were recorded while applying a positive pressure of 1 bar at the back of the nanopipette, which is filled with 0.1 M KCl (without dsDNA) and immersed in 0.1 M KCl with 50% (w/v) PEG 35K[9,10]. As Figure SF7.4a illustrates, we observe a different voltametric response when a positive pressure is applied at the back of the nanopipette. The unique i-V curve associated with the presence of PEG in the external solution with no pressure applied (black curve) is replaced by an i-V similar to the one reported when PEG was absent from the external solution (purple curve). This finding indicates that once an external pressure is introduced $(p_{app} = 1\ bar)$, convective flow of the internal solution pushes PEG molecules away from the nanopipette tip aperture further in the external solution. In contrast, under standard pressure conditions $(p_{app} = 0\ bar)$ PEG molecules are located in close proximity to the nanopipette tip opening, where they interact with ions in the solution influencing the recorded background current. As a result, when single dsDNA molecules pass through the tip aperture, they temporarily displace PEG molecules to complete their translocation to the external solution which causes an interface displacement and ion reorganization leading to the enhanced translocation current peak measure in the presence of PEG. Figure SF7.4b shows the experimental current trace recorded during dsDNA (4.8 kbp) translocation before, during and after the application of pressure $(p_{app} = 1\ bar)$. The translocation current enhancement due to the presence of PEG is completely nullified when the pressure is applied, and it comes back to normal when the pressure is released.

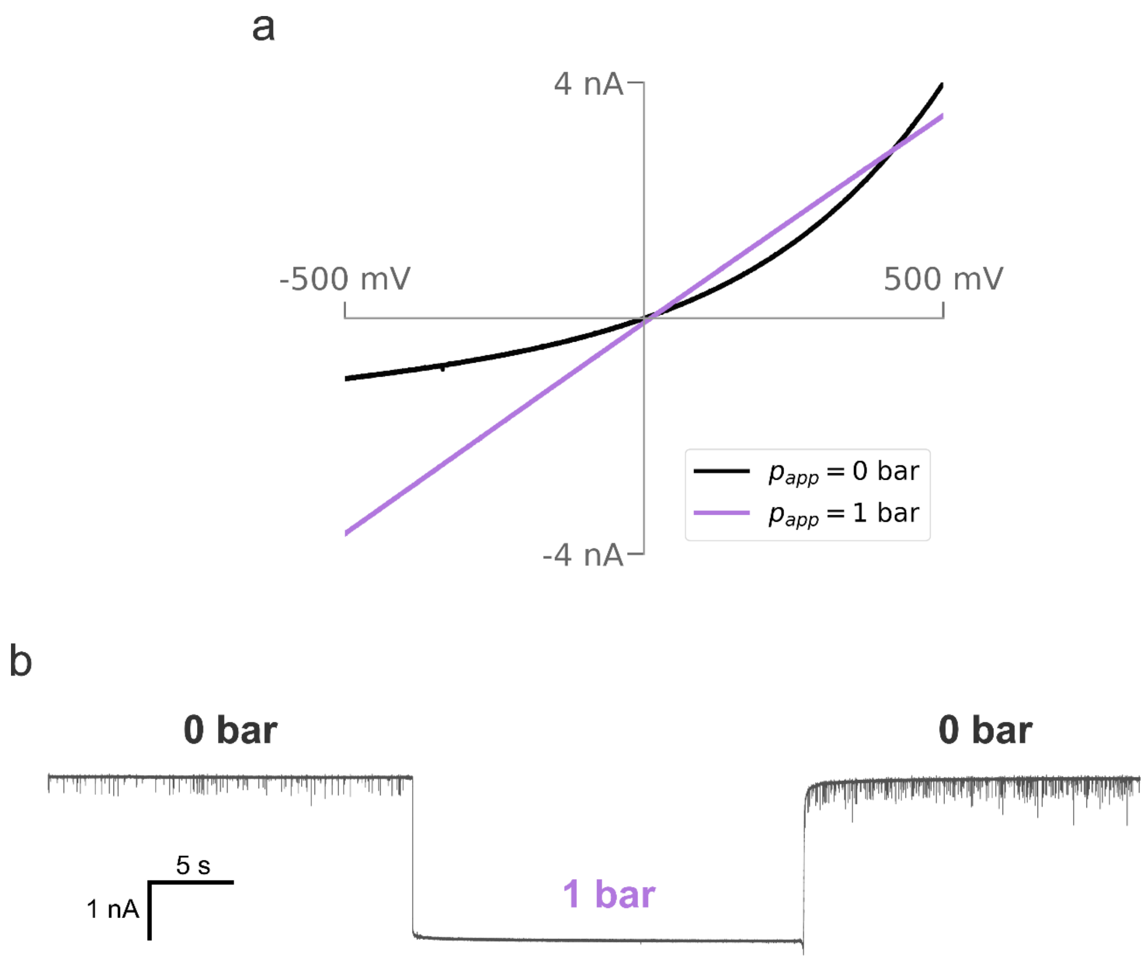

Figure SF7.4 a) Experimental voltammogram for V = ±500 mV, when a pressure of 0 and 1 bar (black and purple curves, respectively) is applied at the back of the nanopipette filled with 0.1 M KCl and immersed in 0.1 M KCl with 50% (w/v) PEG 35K. b) Experimental current trace during 4.8 kbp dsDNA translocation before, during and after the application of 1 bar pressure at the back of the nanopipette for V = -500 mV.

References

(1) White, H. S.; Bund, A. Ion Current Rectification at Nanopores in Glass Membranes. *Langmuir* **2008**, *24* (5), 2212–2218. https://doi.org/10.1021/la702955k.

(2) Perry, D.; Momotenko, D.; Lazenby, R. A.; Kang, M.; Unwin, P. R. Characterization of Nanopipettes. *Anal. Chem.* **2016**, *88* (10), 5523–5530. https://doi.org/10.1021/acs.analchem.6b01095.

(3) Rabinowitz, J.; Edwards, M. A.; Whittier, E.; Jayant, K.; Shepard, K. L. Nanoscale Fluid Vortices and Nonlinear Electroosmotic Flow Drive Ion Current Rectification in the Presence of Concentration Gradients. *J. Phys. Chem. A* **2019**, *123* (38), 8285–8293. https://doi.org/10.1021/acs.jpca.9b04075.

(4) Morgan, H.; Green, N. G. *AC Electrokinetics: Colloids and Nanoparticles*; Research Studies Press, 2003.

(5) *CRC Handbook of Chemistry and Physics, 97th Edition*, 97th edition.; Haynes, W. M., Ed.; CRC Press, 2016.

(6) Zhang, Z.; Ohl, M.; Diallo, S. O.; Jalarvo, N. H.; Hong, K.; Han, Y.; Smith, G. S.; Do, C. Dynamics of Water Associated with Lithium Ions Distributed in Polyethylene Oxide. *Phys. Rev. Lett.* **2015**, *115* (19), 198301. https://doi.org/10.1103/PhysRevLett.115.198301.

(7) Gao, T.; Gao, X.; Xu, C.; Wang, M.; Chen, M.; Wang, J.; Ma, F.; Yu, P.; Mao, L. Label-Free Resistance Cytometry at the Orifice of a Nanopipette. *Anal. Chem.* **2021**, *93* (5), 2942–2949. https://doi.org/10.1021/acs.analchem.0c04585.

(8) Scott, E. R.; White, H. S.; Phipps, J. Bradley. Iontophoretic Transport through Porous Membranes Using Scanning Electrochemical Microscopy: Application to in Vitro Studies of Ion Fluxes through Skin. *Anal. Chem.* **1993**, *65* (11), 1537–1545. https://doi.org/10.1021/ac00059a010.

(9) McKelvey, K.; Edwards, M. A.; White, H. S. Resistive Pulse Delivery of Single Nanoparticles to Electrochemical Interfaces. *J. Phys. Chem. Lett.* **2016**, *7* (19), 3920–3924. https://doi.org/10.1021/acs.jpclett.6b01873.

(10) Lan, W.-J.; Holden, D. A.; Liu, J.; White, H. S. Pressure-Driven Nanoparticle Transport across Glass Membranes Containing a Conical-Shaped Nanopore. *J. Phys. Chem. C* **2011**, *115* (38), 18445–18452. https://doi.org/10.1021/jp204839j.
